# Exploring extra dimensions to capture saliva metabolite fingerprints from metabolically healthy and unhealthy obese patients by comprehensive two-dimensional gas chromatography featuring Tandem Ionization mass spectrometry

**DOI:** 10.1007/s00216-020-03008-6

**Published:** 2020-11-03

**Authors:** Marta Cialiè Rosso, Federico Stilo, Simone Squara, Erica Liberto, Stefania Mai, Chiara Mele, Paolo Marzullo, Gianluca Aimaretti, Stephen E. Reichenbach, Massimo Collino, Carlo Bicchi, Chiara Cordero

**Affiliations:** 1 Dipartimento di Scienza e Tecnologia del Farmaco, Università degli Studi di Torino, Via Pietro Giuria 9, 10125 Torino, Italy; 2 Division of General Medicine, IRCCS Istituto Auxologico Italiano Ospedale S. Giuseppe, 28824 Piancavallo, Italy; 3 Department of Translational Medicine, University of Piemonte Orientale, 28100 Novara, Italy; 4 Computer Science and Engineering Department, University of Nebraska, Lincoln, NE 68588 USA; 5 GC Image, LLC, Lincoln, NE 68508 USA

**Keywords:** Comprehensive two-dimensional gas chromatography-time of flight mass spectrometry, Variable ionization energy, Untargeted fingerprinting by template matching, Saliva metabolome, Fused data from multiplexed ionization

## Abstract

This study examines the information potential of comprehensive two-dimensional gas chromatography combined with time-of-flight mass spectrometry (GC×GC-TOF MS) and variable ionization energy (i.e., Tandem Ionization™) to study changes in saliva metabolic signatures from a small group of obese individuals. The study presents a proof of concept for an effective exploitation of the complementary nature of tandem ionization data. Samples are taken from two sub-populations of severely obese (BMI > 40 kg/m^2^) patients, named metabolically healthy obese (MHO) and metabolically unhealthy obese (MUO). Untargeted fingerprinting, based on pattern recognition by template matching, is applied on single data streams and on fused data, obtained by combining raw signals from the two ionization energies (12 and 70 eV). Results indicate that at lower energy (i.e., 12 eV), the total signal intensity is one order of magnitude lower compared to the reference signal at 70 eV, but the ranges of variations for 2D peak responses is larger, extending the dynamic range. Fused data combine benefits from 70 eV and 12 eV resulting in more comprehensive coverage by sample fingerprints. Multivariate statistics, principal component analysis (PCA), and partial least squares discriminant analysis (PLS-DA) show quite good patient clustering, with total explained variance by the first two principal components (PCs) that increases from 54% at 70 eV to 59% at 12 eV and up to 71% for fused data. With PLS-DA, discriminant components are highlighted and putatively identified by comparing retention data and 70 eV spectral signatures. Within the most informative analytes, lactose is present in higher relative amount in saliva from MHO patients, whereas N-acetyl-D-glucosamine, urea, glucuronic acid γ-lactone, 2-deoxyribose, N-acetylneuraminic acid methyl ester, and 5-aminovaleric acid are more abundant in MUO patients. Visual feature fingerprinting is combined with pattern recognition algorithms to highlight metabolite variations between composite per-class images obtained by combining raw data from individuals belonging to different classes, i.e., MUO vs. MHO.

Graphical abstract

**Electronic supplementary material:**

The online version of this article (10.1007/s00216-020-03008-6) contains supplementary material, which is available to authorized users.

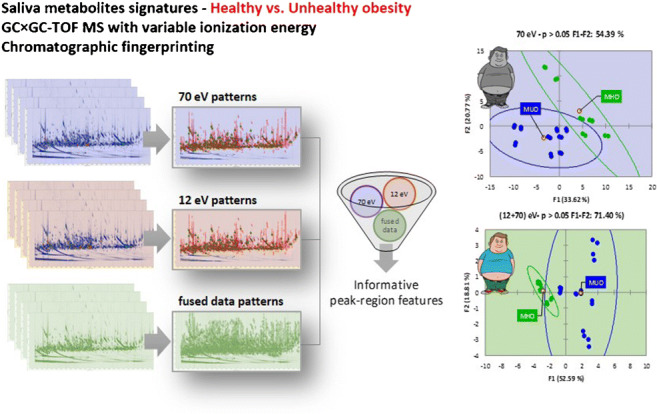

## Introduction

Comprehensive two-dimensional gas chromatography combined with time-of-flight mass spectrometric detection (GC×GC-TOF MS) is a multidimensional analytical platform with great potentials for profiling and fingerprinting of complex samples such as those typically considered in metabolomics studies (e.g., bio-fluids, tissue extracts, and exhaled volatile organic compounds [VOCs]) [1–7]. By this technique, known and unknown analytes (i.e., targeted and untargeted compounds) can be profiled in detail (i.e., detailed profiling) thanks to the great separation power and the high sensitivity achieved by band focusing with thermal modulation [8–11]. Moreover, a new concept of informative fingerprinting can be exploited through the investigation of 2D separation patterns with dedicated algorithms [4, 12]. In this case, chromatographic fingerprinting and pattern recognition provide effective cross-sample analysis and sample-class discrimination, with the intrinsic potential of also using analyte metadata for identification. By this technique, untargeted investigations can be followed by post-targeting to achieve a higher level of information, helping in the interpretation of complex biological phenomena. Human obesity is a particularly suitable clinical condition owing to its multifold association with adverse cardiometabolic events [13].

Mass spectrometry (MS) is a fundamental dimension for any GC×GC platform, especially for complex samples analysis. MS adds another dimension to the system, providing orthogonal information that can be exploited to make fingerprinting more specific, for example by template matching with MS similarity constraints [14], and more accurate when quantitation is performed.

Variable electron ionization (EI) MS systems can increase the dimensionality of the analytical system and thus the specificity of the comparative exploration. Lowering the ionization energy can be done by simply setting the system to operate in such a condition [15] or by patented technologies that enable acquisition by time-switching between two ionization energies. The latter, termed Tandem Ionisation™ [Select-eV™ - US patent number 9,786,480], operates with variable-energy EI across single analytical runs. This patented system adopts, in the ion source, high potentials to accelerate electrons while reducing their energy through the ion chamber [16]. This results in more efficient ionization and reduced loss of sensitivity at low-energy EI with enhanced intensity for structure-diagnostic ions [17].

Recent applications of this technique in the field of motor oils [18] and diesel [19], blood volatiles [20], and food [17, 21] demonstrated that the combination of low ionization energies (10–16 eV) with standard 70 eV provide successful discrimination of samples, with open possibilities to adopt dedicated tandem data processing procedures.

In this study, variable ionization energy TOF MS is explored, for the first time, as an additional dimension of a GC×GC platform in saliva metabolome profiling and fingerprinting. In particular, the differential performance of an untargeted fingerprinting algorithm, based on template matching, is examined by processing single (separate low and high ionization energy) data streams and fused (sum of low and high ionization energy) data streams. The information potential of the system is evaluated through its sensitivity (absolute and relative), response dynamic range, signal response correlation, and fingerprint feature coverage. The challenges provided by this specific application are rooted in the complex metabolic derangement induced by obesity, further complicated by comorbidities occurring in a subset of recruited individuals.

For noninvasive diagnosis and monitoring of local and general diseases, saliva is very accessible and easy to collect. Unlike blood collection, which is almost always uncomfortable and painful for the patient, or feces or urine analysis, which makes people feel embarrassed, saliva collection is highly accessible, by a simple spit into sterile receptacles. Repeated samples can be collected, and diagnosis and monitoring using this method is an innovative and attractive approach [22].

Most notably, saliva metabolites have been noted to parallel metabolic alterations that occur in blood and may thus reflect many pathophysiological and nutritional changes, as well as exposure to medication and environmental factors [23]. Here, we propose to test saliva as a reliable bio-fluid to reveal informative metabolite patterns to determine whether there is a specific salivary signature of obesity per se. There is a limited number of reports on saliva of obese people. Obesity has been demonstrated to lead to a change in the salivary concentration of free sialic acid, total protein, and phosphate as well as activity of peroxidase, which contributes to the formation of dental caries [24]. While previous investigations revealed a reduced antioxidant status and increased proinflammatory cytokine expression in obese individuals, other salivary mediators relevant to obesity could be of metabolic importance for potential clinical and therapeutic implications [25, 26]. However, the majority of obese patients suffer from comorbidities that may modulate saliva secretion either directly or through a required treatment, thus making it difficult to distinguish which changes in salivary parameters could be attributed purely to an increase in body mass. For these reasons, here we focused our analysis on two different populations of subjects with severe obesity (body mass index, BMI ≥ 40 kg/m^2^), according to their metabolic status. Specifically, the recruited subjects were defined as “metabolically unhealthy” obese (MUO) or “metabolically healthy” obese (MHO), depending on whether or not they followed normal metabolic parameters. Generally speaking, a person with MHO has a BMI high enough to be classified as obese but is without the metabolic abnormalities that are usually linked to obesity [27].

## Materials and methods

### Reference compounds and solvents

Pure standards of *n-*alkanes (from *n*-C_7_ to *n*-C_30_) for linear retention index (*I*^*T*^) determination and 1,4-dibromobenzene and 4-fluorophenylalanine for system evaluation and internal standardization were from Merck (Milan, Italy).

The *n*-alkanes mixture was prepared in cyclohexane at a concentration of 100 mg L^−1^; the internal standard (IS) 1,4-dibromobenzene solution was prepared in toluene at a concentration of 10 g L^−1^ and spiked to ready-to-inject derivatized saliva samples at 50 mg L^−1^. The process verification IS, 4-fluorophenylalanine, was prepared in methanol at a concentration of 10 g L^−1^ and spiked to saliva aliquots before the derivatization process at a final concentration of 50 mg L^−1^.

Pure standards for identity confirmation of pyruvic acid, lactic acid, malonic acid, acetoacetic acid, phosphoric acid, succinic acid, glyceric acid, fumaric acid, malic acid, citric acid, alanine (Ala), asparagine (Asn), aspartic acid (Asp), cysteine (Cys), glutamic acid (Glu), glycine (Gln), isoleucine (Ile), leucine (Leu), lysine (Lys), methionine (Met), ornithine (Orn), phenylalanine (Phe), proline (Pro), serine (Ser), threonine (Thr), tryptophan (Trp), tyrosine (Tyr), valine (Val), glycerol, xylitol, mannitol, myo-inositol, fructose, galactose, glucose, saccharose, lactose, and 4-chlorophenylalanine (internal quality control [IQC] for derivatization) were from Merck (Milan, Italy).

Derivatization reagents and HPLC-grade solvents O-methylhydroxylamine hydro-chloride (MOX), N,O-bis(trimethylsilyl)trifluoroacetamide (BSTFA), methanol, pyridine, *n-*hexane, dichloromethane, and toluene were from Merck (Milan, Italy).

### Saliva samples and quality controls

This study includes patients recruited at the Istituto Auxologico Italiano, Verbania, Italy [28, 29]. The height, weight, and waist circumference of participants were measured as a part of regular assessment. Other subjects with body mass index (BMI) of 40 kg/m^2^ or greater were recruited for the study. In total, 34 obese men (BMI ≥ 40 kg/m^2^) were then defined as metabolically unhealthy (MUO, *n* = 24) or metabolically healthy (MHO, *n* = 10), depending on whether their metabolic parameters were within normal ranges, i.e. high fasting triglycerides, 1.7 mmol/L or greater (≥150 mg/dL); reduced HDL cholesterol level (<1.03 mmol/L [<40 mg/dL]); and whether their blood pressure was elevated (≥130 mmHg systolic blood pressure or ≥ 85 mmHg diastolic blood pressure) or on antihypertensive drug treatment and fasting glucose concentration ≥ 5.6 mmol/L (≥100 mg/dL). These criteria were taken from the metabolic syndrome definition [30] and have been used in similar studies. The experimental procedure was approved by the ad hoc Ethical Research Committee of the Istituto Auxologico Italiano (Verbania, Italy). Written informed consent was obtained from the patients. The study protocol conformed to the guidelines of the European Convention on Human Rights and Biomedicine concerning biomedical research.

Saliva from a subset of obese individuals (MHO *n* = 3 and MUO *n* = 5) randomly selected over the available samples and quality controls (QCs) obtained by healthy, normal-weight males (*n* = 4) of the same age were subjected to a standard derivatization protocol [31] adjusted to comply for method sensitivity and metabolite coverage. Sample preparation consisted of the following steps: 100 μL of saliva and a suitable volume of process IS (4-fluorophenylalanine) were carefully mixed (Fisherbrand Whirlimixer vortex; Fisher Scientific, Loughborough, Leicestershire, UK). Then, the solution was dried under a gentle stream of nitrogen before the addition of 25 μL of MOX (20 mg/mL in pyridine) and left to react for 2 h at 60 °C. Next, 75 μL of BSTFA were added, and the mixture was incubated at 60 °C for 1 h. The resulting sample solution was spiked with 1,4-dibromobenzene to a final concentration of 50 mg L^−1^ and diluted in toluene to a final volume of 200 μL. Analyses were immediately run with duplicate injections for each sample; samples were randomized within a 24 h time frame after derivatization.

### GC×GC-TOF MS and Tandem Ionization™: Instrument set-up and conditions

GC×GC analyses were performed on an Agilent 7890B GC unit coupled with a Bench TOF-Select™ system (Markes International, Llantrisant, UK) featuring tandem EI equipped with a SepSolve (Llantrisant, UK) preparation robot with automated tool change model PAL3-RTC. Hard ionization at 70 eV was set for identity confirmation, while 12 eV was applied to explore spectral and response complementarity. The ion source and transfer line were set at 290 °C. The MS optimization option was set to operate in Tandem Ionisation™ with a mass range between 45 and 1000 *m*/*z*; data acquisition frequency was 50 Hz per channel; filament voltage was set at 1.60 V.

The system was equipped with a two-stage KT 2004 loop thermal modulator (Zoex Corporation, Houston, TX, USA) cooled with liquid nitrogen controlled by an Optimode™ V.2 controller (SRA Instruments, Cernusco sul Naviglio, Milan, Italy). The hot-jet pulse time was set at 350 ms; modulation period was 5 s; and cold-jet total flow was progressively reduced with a linear function from 30% of the mass flow controller (MFC) at initial conditions to 8% at the end of the run.

The column set was configured as follows:^1^D DB-5 column (95% polydimethylsiloxane, 5% phenyl; 30 m × 0.25 mm d_c_, 0.25 μm d_f_) coupled with a ^2^D OV1701 column (86% polydimethylsiloxane, 7% phenyl, 7% cyanopropyl; 2 m × 0.1 mm d_**c**_, 0.10 μm d_**f**_) from J&W (Agilent, Little Falls, DE, USA). The first 0.80 m of the ^2^D column, connected in series to the ^1^D column by a silTite μ-union (Trajan Scientific and Medical, Ringwood, Victoria, Australia), were wrapped in the modulator slit and used as a loop capillary for cryogenic modulation. The carrier gas was helium at a constant flow of 1.6 mL min^−1^. The oven temperature program was from 70 °C (2 min) to 120 °C at 10 °C min^−1^, then to 320 °C (1 min) at 4 °C min^−1^.

For saliva samples, 2.0 μL of the derivatized solution (“Saliva samples and quality controls” section) were analyzed under the following conditions: split/splitless injector in split mode, split ratio 1:20, and injector temperature 300 °C. The *n-*alkane liquid sample solution for *I*^*T*^ determination was analyzed under the following conditions: split/splitless injector in split mode, split ratio 1:50, injector temperature 300 °C, and injection volume 1 μL.

### Raw data acquisition and GC×GC data processing

Data were acquired by TOF-DS software (Markes International, Llantrisant, UK) and processed using GC Image GC×GC Software 2.9 (GC Image, LLC, Lincoln, NE, USA). Statistical analysis was performed by XLSTAT 2014 (Addinsoft, New York, NY, USA) and heat map visualization by GENE-E version 3.0.77 (Broad Institute, Inc. Cambridge, MA, USA).

### Method performance parameters: retention times and response repeatability

Method validation was run for 2 weeks in order to evaluate the repeatability of retention times and untargeted and targeted (UT) peak response precision [32]. Retention times in both chromatographic dimensions (^*1*^*t*_*R*_ and ^*2*^*t*_*R*_) were collected from the UT peak regions at 70 eV of analyzed samples [(4 QCs + 8 patients) × 2 process replicates × 2 analytical replicates] for a total of 48 analytical runs processed. Results are reported as % relative standard deviation (% RSD) in Electronic Supplementary Material (ESM) Table S1, with quite good retention time stability, with an average RSD of 0.20% for ^*1*^*t*_*R*_ and 1.90% for ^*2*^*t*_*R*_*.* Response repeatability was calculated on 552 reliable peaks from fused data and was based on normalized responses from QC samples (4 QCs × 2 process replicates × 2 analytical replicates = 16 analytical runs) acquired over 2 weeks. As reported in ESM Table S1, repeatability % RSD averaged 12.13, with a minimal value of 3.28 for peak feature #134 and a maximum of 49.99 for #31.

### UT fingerprinting workflow

**Fig. 1:**
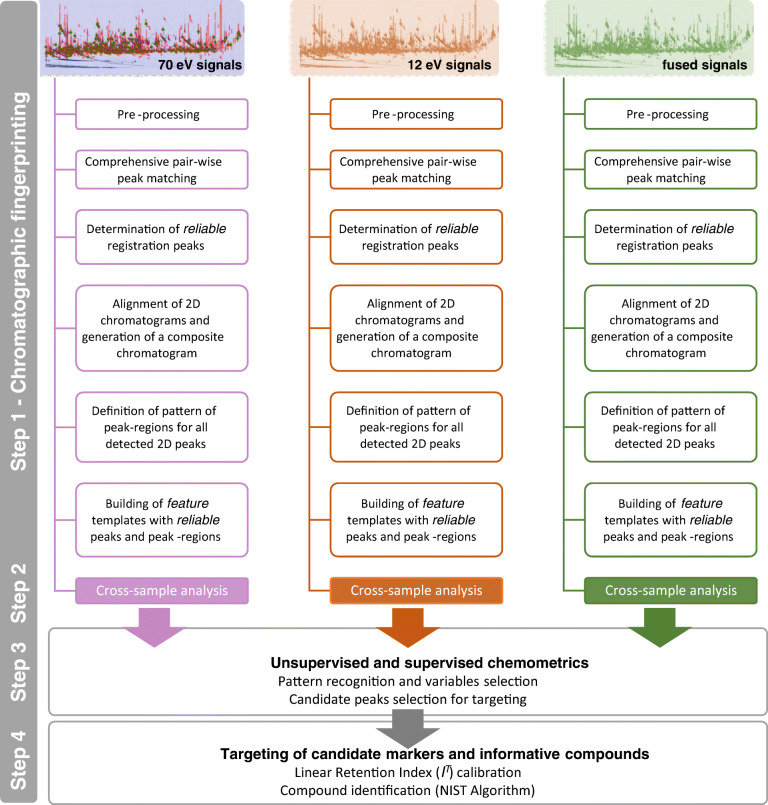
2D data elaboration workflow based on untargeted fingerprinting on single (12 and 70 eV) and fused (12 + 70 eV) data streams

The 2D data elaboration workflow is illustrated in Fig. 1 and detailed in the ESM Fig. S1. It is based on an approach developed to comprehensively map UT features (i.e., peaks and peak regions) for the most inclusive fingerprinting [33, 34]. In this specific application, the pre-targeting of analytes was skipped by performing fully automated untargeted fingerprinting on parallel data streams generated by Tandem Ionization™ (70 and 12 eV channels). In addition, composite chromatograms [17, 19] were computed by summing the parallel 12 and 70 eV data streams for each single analytical run, and submitted to the UT processing workflow.

The template-matching strategy [14] is at the basis of the comprehensive realignment of untargeted features across all chromatograms. The metadata corresponding to all detected 2D peaks and peak regions (retention times, MS fragmentation patterns, and detector responses) are actively used to establish reliable correspondences between chemical entities across multiple chromatograms. The specificity of the approach relies on the constraints applied to validate positive matches. Based on previous studies aimed at validating the robustness and the accuracy of the process [17, 35], a spectral-similarity threshold of 750 was imposed for both direct match factor (DMF) and reverse match factor (RMF) between *reference* (template) and *analyzed* (candidate) MS signatures using the NIST MS Search algorithm, version 2.0 (National Institute of Standards and Technology, Gaithersburg, MD, USA) [35]. Matching was between “peak spectra,” i.e., the MS signature from the largest data point within the peak, and a signal-to-noise (S/N) threshold of 100 was fixed to discard 2D peaks with inconsistent MS information.

The output of the full untargeted fingerprinting is a data matrix of aligned 2D peaks and peak regions together with related metadata (^1^D and^2^D retention times, MS fragmentation pattern, base peak and molecular ion *m*/*z*, and total ion current [TIC] response), which is available for further processing [36–38]. Here, there are three independent data matrices, for the 70 eV, 12 eV, and fused data streams.

The untargeted fingerprinting (Step 1 in Fig. 1) was performed automatically by GC Image Investigator™ version 2.9 software (GC Image, LLC, Lincoln, NE, USA) to generate peak and peak-region features [12, 39]. For this purpose, peak detection thresholds were set according to absolute responses (TIC counts) corresponding to S/N threshold of 100 (i.e., 500,000 counts/70 eV; 5000 counts/12 eV; 100,000 counts/fused data). Values were confirmed by visual supervision to yield comparable 2D peak detection across channels. The process [4, 12, 33, 38–40] aligned the 48 chromatograms [(4 QCs + 8 patients) × 2 process replicates × 2 analytical replicates] for each data stream (12 and 70 eV) plus the fused data (12 + 70 eV) using a set of registration peaks, named *reliable* peaks, to produce a composite chromatogram from which peak regions were extracted. Reliable peaks are those 2D peaks that were positively matched across more than half of the chromatograms (i.e., 25 of the 48 sample chromatograms).

**Fig. 2:**
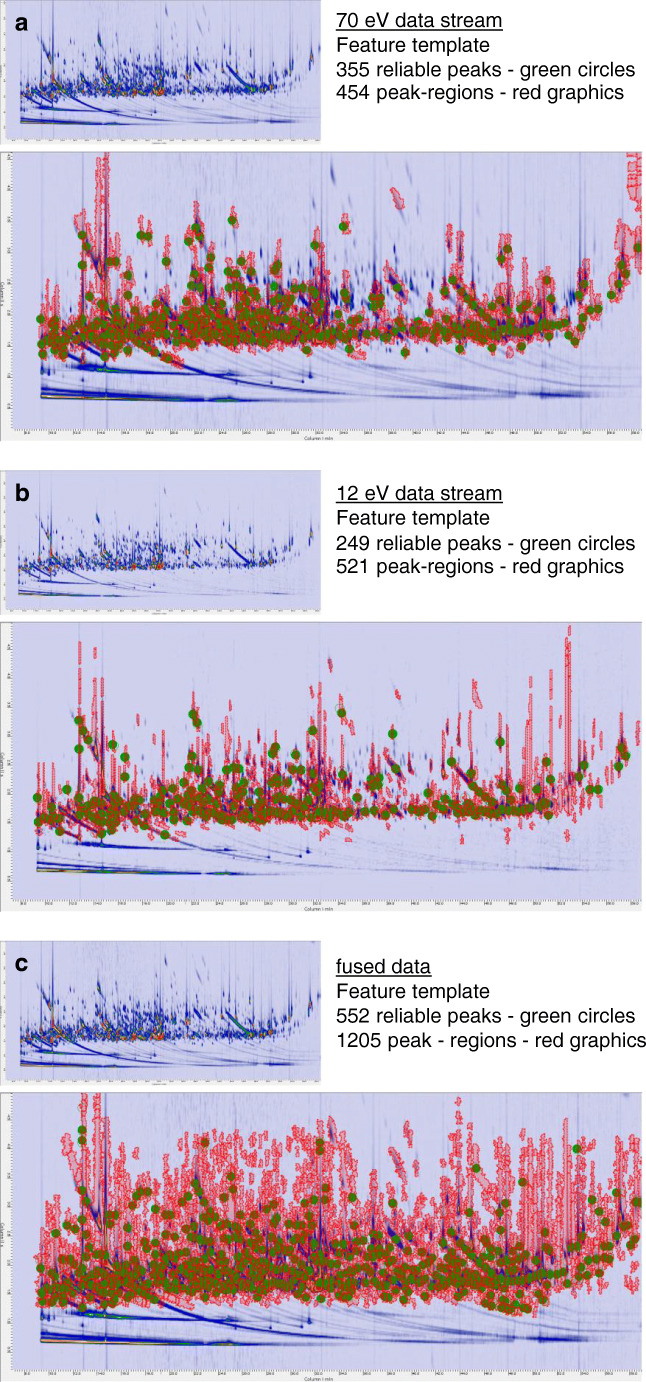
Pseudo-color chromatographic images corresponding to an unhealthy obese (MUO) patient, resulting from the 70 eV ionization channel (**a**), 12 eV (**b**), and after fusion of the tandem data streams (**c**). Green circles indicate reliable 2D peaks, and red graphics indicate untargeted peak regions. Details on feature template objects are also reported

The resulting templates with untargeted (reliable) peaks and peak regions extracted by the composite chromatograms (i.e., the feature templates) are shown in Fig. 2 where the saliva metabolome signature of an unhealthy obese (MUO) patient is shown as it results from the 70 eV ionization channel (2a), 12 eV (2b), and after fusion of the tandem data streams (2c). The visual complexity of the templates is indicative of the richness of the untargeted fingerprint. Details on the results of this processing are discussed in “Full untargeted fingerprinting on single and fused data streams” section.

Targeting of informative compounds and potential markers (Step 4 in Fig. 1) was performed for those peak regions that demonstrated higher information potential by unsupervised and supervised chemometrics (Step 3 in Fig. 1) for discriminating healthy from unhealthy obese saliva signatures. Identification of targeted analytes was carried out by matching candidate EI-MS fragmentation patterns at 70 eV (NIST MS Search algorithm, version 2.0) with those collected in commercial and in-house databases (subject to a DMF threshold of 900 and RMF threshold of 950). In addition, linear retention indices (*I*^*T*^) were adopted as a further constraint; experimental values were compared with NIST reference indices using a tolerance of ±10 units.

## Results and discussion

In this study, the complex saliva metabolome was explored, for the first time, by combining the separation power of GC×GC with the complementary information provided by TOF MS with variable ionization energy. The fingerprinting process, based on pattern recognition (i.e., UT fingerprinting), was applied in its full flexibility to cross-align features (2D peaks and peak regions) between tandem signals and with the fused data, to validate the role of informative analytes and to facilitate their identification using the 70 eV spectra.

The next sections discuss full untargeted fingerprinting results and performance obtained by independent processing of single (70 and 12 eV) and fused (70 eV + 12 eV) data streams as illustrated at Steps 1 and 2 in Fig. 1).

### Full untargeted fingerprinting on single and fused data streams

The sample set under consideration in this study exhibited several analytical challenges related to both the inter-individual variability of the saliva metabolome [41] and its intrinsic complexity due to the concurrent presence of comorbidity and metabolic derangements in obese patients. In such a context, the combination of two ionization energies was beneficial due to their different and complementary dynamic ranges of response [19–21, 42]. It was demonstrated by previous investigations that when operating at lower ionization energies (e.g., 10–16 eV), the generalized reduction in analyte absolute response was accompanied by higher relative sensitivity, expressed as signal-to-noise ratio (S/N), for several analytes [17, 21]. This higher relative sensitivity was much more evident for those analytes that showed the most dissimilar fragmentation patterns between tandem channels.

These benefits were exploited to delineate metabolite signatures capable of discriminating healthy from unhealthy obese patients. Of course, this preliminary study is just a proof of concept useful for evaluating the potential and the synergism of adding extra dimensions at the MS detection level in challenging scenarios. The limited number of samples available does not allow definitive generalized conclusions regarding metabolomics; however, the fine-tuning of 2D data processing strategies and the critical evaluation of the actual performance of different workflows will inform future investigations with larger sample sets.

The 2D data processing strategy was conducted in a full untargeted fashion, at the first stage. For a better understanding of the signal characteristics, here follows some details on: (a) background signal intensity before and after noise subtraction [43]; (b) number of detected 2D peaks above a fixed threshold; and (c) range of variation for S/N and 2D peak absolute response. All these data are summarized in Table 1 and refer to average values computed from the MUO #20 patient saliva replicates (*n* = 4).

**Table 1:** Data processing parameters based on single (12 and 70 eV) and fused (12+70 eV) data streams. Untargeted fingerprinting statistics and supervised chemometrics are detailed throughout the text

Parameter	Detection channel		
	70 eV	12 eV	Fused data
Average background intensity (counts) (retention window^1^D 15.5–19.0 min /^2^D 4.5–5.0 s)			
Before noise subtraction	23,090	1840	26,140
After noise subtraction	1150	10	1140
No. detected 2D peaks S/N ≥ 100	517	336	549
Absolute response TIC (peak volume)			
Min	5.17E+05	2.63E+04	1.08E+05
Max	1.69E+08	4.59E+07	2.12E+08
RSD	174%	319%	198%
Signal-to-noise ratio (S/N) TIC			
Min	100	100	100
Max	59,007	36,209	52,954
RSD	251%	206%	226%
Fingerprinting and statistics			
Feature template			
No. reliable peaks	355	249	552
No. peak regions	454	521	1205
% Matched peaks on cumulative chromatogram	99%	97.6%	95.3%
No. 2D peaks S/N ≥ 100	1954	1729	2267
Supervised statistics			
Kruskal–Wallis—Dunn’s test and Bonferroni correction			
Peak regions meaningful *p* (alpha 0.05)	100	110	135

Results confirm that at lower ionization energies, the total signal intensity is lower (1840 vs. 23,090 counts) by one order of magnitude, while for the fused data stream, the average background level is close to that at 70 eV (26,140 vs. 23,090 counts). After noise subtraction, applied here according to the Sternberg algorithm [44], the resulting baseline was much more effectively reduced at 12 eV, where the reduction was of two orders of magnitude. This operation did not affect spectral quality, as demonstrated by the number of reliable peaks detected (see below).

The number of detected 2D peaks above the fixed TIC response thresholds (“UT fingerprinting workflow” section) and S/N ≥ 100 was comparable between 70 eV and the fused data streams, with the latter exceeding the reference channel (i.e., 70 eV) by 6%. On the other hand, as expected, at lower energies, 2D peaks were 36% lower than those at 70 eV. Absolute response values, calculated on the TIC channel, span three orders of magnitude following a log-normal distribution (data not shown), but with a wider range of variation calculated for the 12 eV channel: % relative standard deviation (RSD) of 319% vs. 174% at 70 eV. This signal characteristic is of great interest because it enables exploration of complex fractions with a wider dynamic range. Analyte response/concentration variations can be more effectively captured, especially on the higher range, at higher intensity values, where at 70 eV detector saturation may easily occur. It should be stressed that with band compression in space of thermal modulation, the analyte concentration gradient entering the ion source and then reaching the detector is higher than with conventional 1D GC-MS. The fused data stream, combining 70 and 12 eV signal characteristics, span a wider range of responses compared to the 70 eV with larger RSD.

The impact of signal characteristics can be appreciated by untargeted fingerprinting processing (“UT fingerprinting workflow” section). The single and summed (i.e., fused) data streams were independently processed by Image Investigator software (GC Image, LLC), applying the most relaxed conditions [39] for reliable peak selection. By this approach, untargeted 2D peaks that are matched (subject to retention time constraints and 750 MS similarity) across at least half of the chromatograms of the set (>24 of the 48 individual chromatograms) are recorded and adopted as alignment points for the construction of a composite image (ESM Fig. S2) for each channel set. Results are summarized in Table 1 and discussed in the following text.

According to signal characteristics, the number of reliable peaks at 70 eV was higher than at 12 eV (355 vs. 249); this result is in line with absolute response data and relative sensitivity (S/N range), confirming that at higher ionization energies, the number of 2D peaks with consistent spectral information is larger. For the fused data stream, the number of reliable peaks was 55% greater than for 70 eV, indicating the benefits of adding low-ionization spectral information to the processing. At lower energies, the fragmentation is limited, and fragments with larger *m/z* values prevail [17, 21], with benefits for spectral interpretation [45]. Spectral dissimilarity and fragmentation pattern characteristics are discussed in more detail for analytes of interest in this application in “Informative metabolites and their response from tandem signals” section.

The benefits of data fusion also are reflected in the number of untargeted peak regions delineated over the composite chromatogram(s) obtained by realignment and summing of single or fused data images for the 48 runs. Peak regions correspond to all detected 2D peaks above the TIC response fixed thresholds (i.e., 500,000, 70 eV; 5000, 12 eV; 100,000, fused data). Single ionization channels had almost the same coverage by mapping 454 and 521 untargeted peak regions at 70 and 12 eV, respectively, whereas the fused data doubled the feature coverage, reaching 1205 peak regions. This result emphasizes the advantages of processing hybrid data, exploiting in full the potential benefits of the tandem channels.

Last but not least, the feature template created from fused data has a greater compositional coverage. For the single ionization channels, the reliable peaks, with 355 at 70 eV or 249 at 12 eV, achieved a 99% and 98% matching rate on the corresponding composite chromatograms, but covered only 18% and 14% of the detected 2D peaks. For fused data, the 552 reliable peaks achieved a 95.3% matching rate and covered 24% of the detected peaks.

The next section discusses results for unsupervised and supervised statistical analyses of the saliva patterns resulting from untargeted fingerprinting for the different detection channels. 2D peaks and peak regions with meaningful variations are then examined to determine a putative identity and hypothesize an informational role.

### Chromatographic feature selection and results cross-validation

**Fig. 3:**
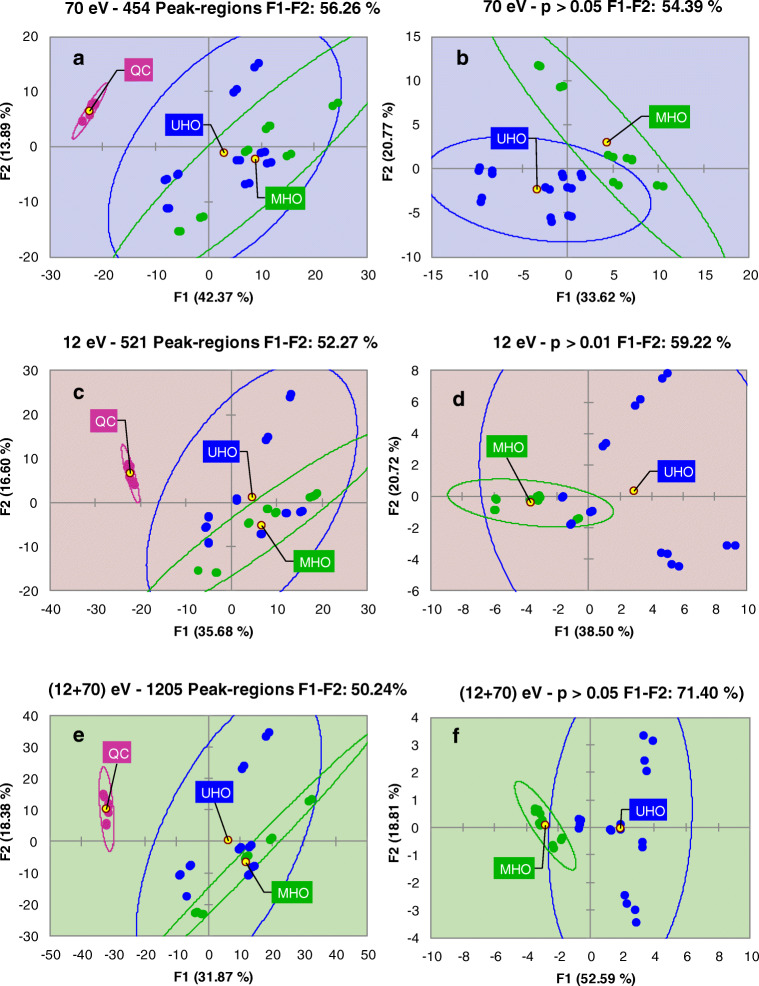
Principal component analysis (PCA) including all analyzed samples (48 runs) and based on % response (TIC 2D volumes) of realigned 2D peak regions from the feature template: **a** 70 eV, **c** 12 eV, **e** fused data streams. QC samples are retained to show response consistency and to illustrate the statistical distance between the saliva of healthy subjects (QCs) and that of MUO/MHO patients. Feature reduction by the Kruskal–Wallis test with post hoc correction by Dunn’s test and Bonferroni correction results in PCA loading plots of **b** (70 eV), **d** (12 eV), and **f** (fused data streams)

As a first unsupervised exploration, principal component analysis (PCA) was conducted for all analyzed samples (48 runs) using % response (based on TIC 2D volumes) for realigned 2D peak regions from the feature template. At this stage, QC samples were included to provide a first impression of the overall quality of the responses and the degree of overlap between saliva metabolome of obese patients and that of healthy individuals. Results are visualized in Fig. 3. For all detection channels (Fig. 3a, c, e), QCs are separately clustered with confidence ellipses (95% of confidence), indicating response stability. At the same time, saliva from obese patients shows a certain degree of overlapping, in line with the common metabolic traits expected for this group. However, the saliva metabolic signature from unhealthy obese patients (UHO, blue circles) appear to be affected by a greater variability, probably due to the presence of comorbidities.

PCA score plots based on the % response data from single (Fig. 3a and c) or fused data (Fig. 3e) are highly concordant, although with 70 eV response data the total explained variance reached the highest value (i.e., F1 and F2, 56.3%). The combination of results from three independent elaborations takes advantage of the complementary nature of tandem signals that enables effective cross-validation.

The next step included a feature selection by removing from the computation 2D peak regions whose variation between UHO and MHO groups was not meaningful in the description of the phenomenon. Due to the feature response distributions, which are not normal, a nonparametric test was applied, i.e., Kruskal–Wallis, with post hoc correction by Dunn’s test and Bonferroni correction. Peak regions with meaningful *p* values (alpha 0.05) were kept for further processing.

Results are visualized in Fig. 3b d, f for 70 eV, 12 eV, and fused data, respectively. The selection of variables had almost the same results in terms of retained variables (on average 100 peak regions, Table 1) but with different outcomes on the total explained variance. Fused data achieved 71% of the total variance explained by the first two components, with also an almost independent clustering of the groups of patients.

For 12 eV, the UHO samples were more dispersed, suggesting that at lower energies, the wider dynamic range of responses, although beneficial for more accurate profiling of metabolite response variations, confounds intra-class variability.

Results for fused data streams showed the highest explained variance by the first two PCs and were further explored by supervised statistics (i.e., partial least squares discriminant analysis [PLS-DA] and variable importance in projection [VIP]) to rank and select untargeted peak region features with a role in the discrimination between UHO and MUO saliva signatures and to focus identification efforts on those potential markers.

### Informative metabolites and their response from tandem signals

Supervised statistics by PLS-DA and VIPs highlight discriminant peak regions within those preselected at the previous step. Features with VIPs ≥1 are listed in Table 2 together with retention times in the two chromatographic dimensions (^*1*^*t*_*R*_; ^*2*^*t*_*R*_), estimated linear retention index (*I*^*T*^) and literature reference (NIST library [46]), putative identification (criteria in “UT fingerprinting workflow” section), and % of absolute response variation between samples groups (MUO vs. MHO).

**Table 2:** List of discriminant features together with their identifiers over the 2D space (#peak region and #reliable peak), retention times (^*1*^*t*_*R*_; ^*2*^*t*_*R*_), experimental and tabulated linear retention index (*I*^*T*^), putative identification, % difference between unhealthy (MUO) and healthy (MHO) obese patients. PLS-DA VIP values and relative standard deviation (SD) are also reported

#Peak region	#Reliable peak	^*1*^*t*_*R*_ (min)	^*2*^*t*_*R*_ (sec)	*I*^*T*^	Lit *I*^*T*^	Compound name	HMDB #	% Diff	VIP value	SD
306-140	306	33.25	1.70	1923	1921	D-(+)-Glucuronic acid γ-lactone, 3TMS methyloxime (anti)	0006355	325.2	1.35	0.31
351-10	351	13.92	2.98	1192	1249	Urea, 2TMS	0000294	99.0	1.20	0.24
129-97	129	24.42	1.62	1589	1569	2-Deoxy-D-ribose, 3TMS methyloxime (syn)	0003224	87.8	1.19	0.14
101-428	101	42.42	2.38	2270	NA	N-Acetylneuraminic acid methyl ester, 5TMS	0000796	77.2	1.21	0.37
32-4	32	25.17	1.84	1624	NA	5-Aminovaleric acid, 3TMS	0003355	75.3	1.36	0.20
282-172	282	36.58	2.24	2052	2061	N-Acetyl-D-glucosamine, 4TMS methyloxime (anti)	0000215	53.9	1.58	0.22
33-18	33	10.50	1.76	1052	1048	3-Methyl-2-oxobutanoic acid, TMS	0000019	52.7	1.27	0.39
19-134	19	39.25	2.86	2149	2146	N-Acetyl-D-glucosamine, 4TMS trimethylsilyloxime (isomer II)	0000215	48.8	1.42	0.21
106-96	106	53.24	1.70	2678	2661	Sucrose, 8TMS	0000258	40.9	1.24	0.41
326-234	326	17.58	1.88	1330	1270 (DB-1)	Phenoxyethanol, TMS	0041607	36.8	1.27	0.41
79-131	79	40.50	1.68	2198	2246 (DB-1)	Stearic acid, TMS	00827	28.2	1.29	0.31
213-57	213	36.67	2.12	2058	2068	N-Acetyl-D-glucosamine, 4TMS methyloxime (syn)	0000215	26.1	1.41	0.27
54-47	54	24.83	1.84	1574	1586	2-Hydroxyglutaric acid, 3TMS	0000606	20.6	1.22	0.35
47-22	47	27.83	1.64	1718	1712	D-Rhamnose, 4TMS methyloxime (anti)	0000849	13.5	1.28	0.47
376-43	376	31.67	1.60	1863	1859	D-Fructose, 5TMS methyloxime (anti)	0000660	13.0	1.19	0.44
10-55	10	30.42	1.84	1836	1845	Citric acid, 4TMS	0000094	10.8	1.16	0.33
37-11	37	50.67	1.84	2582	2607	Glycerol monopalmitate, 2TMS	0011564	9.8	1.16	0.38
364-60	364	31.42	1.88	1854	NA	N-α-Acetyl-L-lysine, 3TMS	0000446	4.9	1.15	0.32
27-111	27	32.25	1.64	1885	1883	D-Glucose, 2,3,4,5,6-pentakis-O-(trimethylsilyl)-, o-methyloxyme, (1Z)-	0000122	−4.4	1.16	0.29
96-64	96	32.33	2.16	1889	1883	Tyrosine, 2TMS derivative	0000158	−12.6	1.17	0.39
31-2	31	14.58	2.08	1217	1286	Phosphoric acid, 3TMS	0001429	−13.7	1.17	0.35
51-109	51	22.33	2.42	1510	1522	Pyroglutamic acid, 2TMS	0000267	−17.8	1.19	0.23
50-192	50	50.08	1.78	2560	2587	Lactulose, 8TMS (isomer I)	0000740	−40.0	1.29	0.40
104-83	104	18.65	1.68	1367	1368	Serine, 3TMS	0000187	−59.4	1.30	0.24
28-340	28	33.17	1.58	1920	1920	D-Sorbitol, 6TMS	0000247	−71.9	1.18	0.23
265-178	265	51.25	2.22	2604	2617	D-Lactose, (isomer 2), 8TMS	0041627	−87.5	1.38	0.29
183-160	183	51.33	1.80	2526	2529	D-Lactose, (isomer 1), 8TMS	0041627	−87.5	1.35	0.28
181-230	181	52.50	1.80	2666	2675	D-Lactose, octakis(trimethylsilyl) ether, methyloxime (isomer II)	0041627	−93.8	1.29	0.31
195-372	195	51.75	1.86	2653	2659	D-Lactose, octakis(trimethylsilyl) ether, methyloxime (isomer I)	0041627	−96.9	1.40	0.29

*HMDB* Human Metabolome Database

Within targeted analytes, those with the larger variations were D-(+)-glucuronic acid γ-lactone (i.e., glucuronolactone +325.2%), N-acetyl-D-glucosamine (+128.8%), urea (+98.0%), 2-deoxy-D-ribose (+87.8%), N-acetylneuraminic acid methyl ester (+77.2%), 5-aminovaleric acid (+75.3%), 3-methyl-2-oxobutanoic acid (+52.7%), and sucrose (+40.9%). All of them were upregulated in unhealthy obese patients with meaningful variations as indicated by VIP values (Table 2). On the other hand, the saliva metabolome of healthy obese individuals was connoted by a higher relative abundance of D-lactose (+365.0%), serine (+59.4%), and pyroglutamic acid (i.e., indicating L-proline and L-glutamine, +17.8%).

**Fig. 4:**
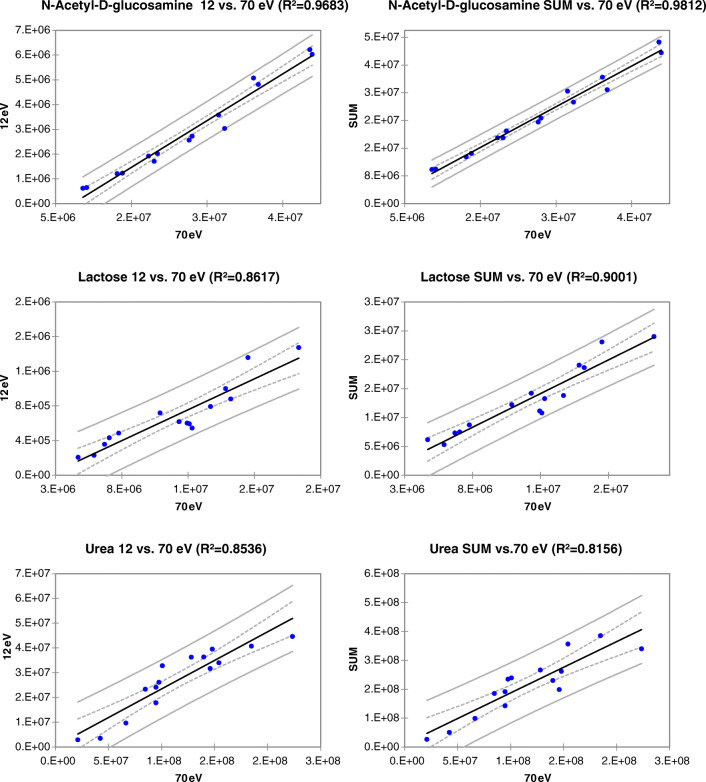
Linear regression results for discriminant analytes (i.e., N-acetyl-D-glucosamine, lactose, and urea) on the basis of TIC response data at 12 eV (left side) and from fused signals (12 + 70 eV − SUM − right side) compared to 70 eV taken as reference (independent variable − *x*)

Discriminant peak-region TIC responses were examined to evaluate the degree of correlation between tandem signals. Figure 4 shows linear regression results for N-acetyl-D-glucosamine, lactose, and urea on the basis of TIC response at 12 eV (left side) and from fused signals (12 + 70 eV − SUM − right side) compared to 70 eV taken as reference (independent variable − *x*). Results are satisfactory: all determination coefficients (R^2^) are above 0.800, indicating that tandem signals are highly consistent and that their combination, by fused data streams or by independent processing, might be beneficial for the dynamic range of the method.

**Fig. 5:**
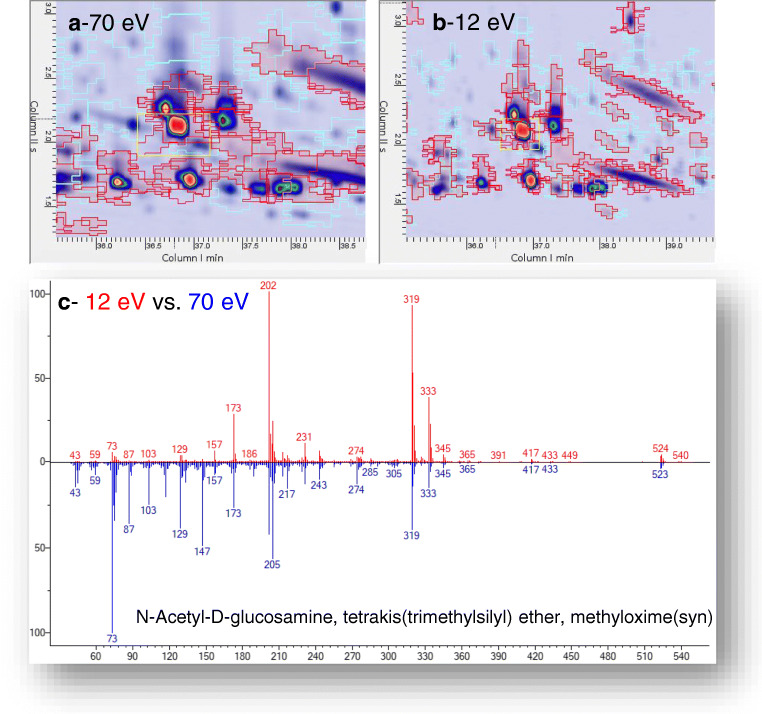
Enlarged areas of the contour plots corresponding to the elution region of a N-acetyl-D-glucosamine isomer: **a** 70 eV ionization; **b** 12 eV energy; **c** head-to-tail comparison of the resulting fragmentation pattern between the two channels

The differential sensitivity between the two channels (70 and 12 eV) and the dissimilarity between fragmentation patterns is illustrated in Fig. 5 for one of the derivatization isomers of N-acetyl-D-glucosamine and in ESM Fig. S3 for a selection of metabolites covering different chemical classes. With image colorization, the 70 eV channel (Fig. 5a) shows highest absolute response compared to 12 eV (Fig. 5b), while spectra (Fig. 5c) mainly differ for the presence of lower fragments at 70 eV (73, 129, 147 *m*/*z*) that are less represented at lower energy, in favor of larger fragments with a higher intrinsic informative role (202, 319, 333 *m*/*z*).

**Fig. 6:**
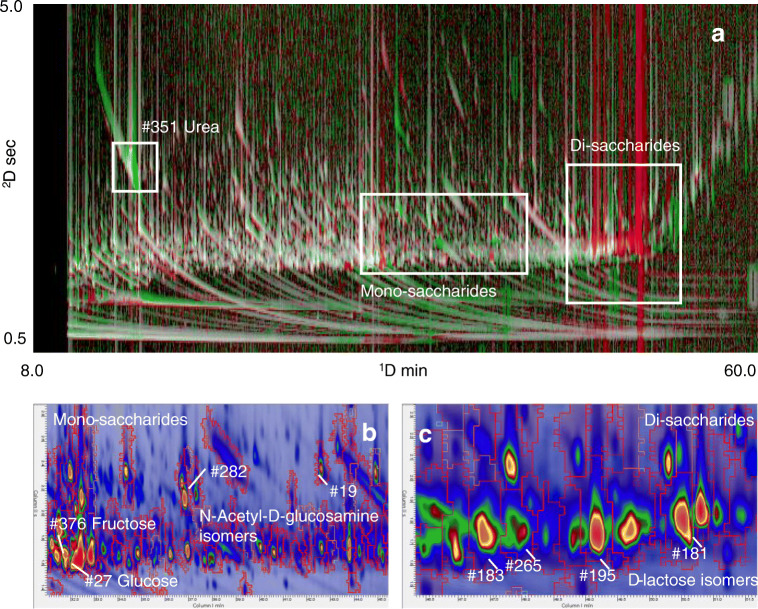
Colorized fuzzy ratio rendering (**a**) of the differential response between composite class images from MUO (*analyzed* image) and MHO (*reference* image) samples. Areas in the pattern colored in red correspond to features whose relative TIC response (normalization is done on the total image response) is higher in the *reference* class image (i.e. MHO), while areas in green represent features with higher responses in the *analyzed* class image (i.e., MUO). Monosaccharide (**b**) and disaccharide (**c**) regions are detailed in enlarged areas

The interindividual variations in the salivary metabolome, due to several confounding variables that might have a more decisive impact on the distribution of metabolites, can be minimized by applying data processing strategies that accumulate aligned data point features (i.e., TOF MS detector events) to generate composite class images [47] for pair-wise comparisons. Based on the fingerprinting workflow illustrated in Fig. 1, composite class images representing the salivary metabolite signatures of healthy (MHO) and unhealthy (MUO) obese patients were built from fused data images. In this way, comparative visualization [12] was adopted to highlight pattern variations between groups. Figure 6a shows a colorized fuzzy ratio rendering [40] that illustrates the differential response between MUO (*analyzed* image) and MHO (*reference* image) composites. Areas in the pattern colored in red correspond to components whose relative TIC response (normalization is done on the total image response [40]) was higher in the *reference* class image (i.e., MHO), whereas areas in green are components with higher responses in the *analyzed* class image (i.e., MUO). Except for urea, eluting early in the chromatogram (peak region #351) and present in higher amounts in unhealthy obese patients, there is a clear pattern of upregulated di-saccharides at higher retention indexes (Fig. 6c) that were putatively identified as lactose isomers. On the other hand, among monosaccharides (Fig. 6b), the trends for fructose (#376) and glucose (#27) are in contrast—see green and red colorization, respectively—while N-acetyl-D-glucosamine isomers (#19 and #282) were clearly upregulated in MUOs. Univariate statistics on analytes’ relative distributions in sample classes, by box-plot visualization, is provided as ESM Fig. S4.

### Insights on saliva metabolite differential signatures

With respect to biological interpretation of the analytical data, although sampling was limited to a few subjects within all recruited patients in the global study, it appears that saliva of metabolically unhealthy obese patients had a higher relative abundance of N-acetyl-D-glucosamine. This amide derivative of glucose in mammals is generally bonded to serine or threonine residues of a protein, giving the O-GlcNAcylation. O-GlcNAcylation appears to have a role in autophagy, insulin signaling, and stress response [48], while large amounts of O-GlcNAcylation resulted in insulin resistance in adipocytes [49]. The MUO population, when profiled by multiplexed lectin microarray for carbohydrates present on proteins, resulted in clear and distinctive patterns of GlcNAcylation [28].

Urea, another informative analyte from the fingerprinting analysis, is the principal carrier of waste nitrogen derived from the protein catabolism. High levels of salivary urea were found to be abundant in chronic kidney disease patients [50], while higher D-(+)-glucuronic acid γ-lactone amounts were found in patients with hepatic steatosis [51]. On the other hand, 5-aminovaleric acid (+75.3% in MUO), a lysine degradation product, is a normal metabolite present in human saliva, with a tendency to elevated concentration in patients with chronic periodontitis. Bacterial contamination and decomposition of salivary proteins is primarily responsible for elevated salivary levels of this metabolite [52].

Data on saliva level/concentration of lactose are lacking, although evidence of urine excretion of this metabolite is correlated to milk consumption [53] and, in its turn, also related to fermentation by gut microbiota that in obesity has a great impact on metabolic homeostasis [54].

When compared with QC patterns, obtained from healthy male subjects, both MHO and MUO were characterized by higher levels of butyric acid and myo-inositol, previously confirmed to be obesity markers found in blood and urine [55, 56].

The role of dietary carbohydrates in the development and maintenance of obesity is a topic that is receiving increasing attention [57]. Glucose is catabolized via glycolysis to pyruvate, which under aerobic conditions, is converted into acetyl coenzyme A, the entry point into the tricarboxylic acid (TCA) cycle. Under anaerobic conditions, pyruvate is instead converted into lactate by lactate dehydrogenase. In obesity, the levels of glucose, lactic acid, fructose, glycerol, mannose, sorbitol, xylose, gluconic acid, and glucuronic acid are increased [58, 59].

Overall, preliminary results from saliva signatures from obese patients indicate a hitherto unrecognized and fairly novel role for carbohydrate and amino acid metabolism in obesity while suggesting that altered metabolic fingerprints may contribute to a better understanding of the pathogenesis of different clinical contexts linked to obesity, specifically MHO vs. MUO, but also to better elucidate the inflammatory milieu detectable in the saliva of MUO, which could help to better prevent many obesity-associated pathological conditions, including cardiovascular and metabolic diseases [28].

## Conclusions

Comprehensive two-dimensional gas chromatography, when combined with Tandem Ionization™ TOF MS, improves confidence in untargeted fingerprinting of complex samples. Chromatographic fingerprints from saliva metabolites obtained by summing raw MS data acquired at two ionization energies (i.e., 12 and 70 eV) had a larger number of reliable features (i.e., 2D peaks reliably matched across chromatograms), indicating a higher spectral consistency and stability. Low-ionization energy data showed a wider dynamic range of responses and good correlation with 70 eV response. Moreover, lower ionization limited fragmentation by producing MS spectral signatures dominated by medium-to-high *m*/*z* fragments with an intrinsically higher information role on analyte identity.

Although the very limited number of samples does not allow conclusive results, analytes discriminating saliva signatures of MUO vs. MHO were putatively identified. Their information role might be now be placed in the more general context of metabolic derangement caused by obesity and concurrent alterations due to comorbidities. The study provides a proof of concept on the advantages of adding extra dimensions to the analytical system adopted for chromatographic fingerprinting of biological fluids; it delineates an effective workflow that can be adopted with a larger data set.

## Electronic supplementary material

ESM 1 (PDF 2.03 mb)
