# Supplementary material for: Exploring extra dimensions to capture saliva metabolite fingerprints from metabolically healthy and unhealthy obese patients by comprehensive two-dimensional gas chromatography featuring Tandem Ionization mass spectrometry
Source: Anal Bioanal Chem. 2020 Nov 3;413(2):403–18. doi: 10.1007/s00216-020-03008-6 (PMC7806578; doi:10.1007/s00216-020-03008-6)
Supplement: Supplementary file 1 — (PDF 2.03 mb) [file 216_2020_3008_MOESM1_ESM.pdf]

Analytical and Bioanalytical Chemistry

Electronic Supplementary Material

Exploring extra dimensions to capture saliva metabolite fingerprints from metabolically healthy and unhealthy obese patients by comprehensive two-dimensional gas chromatography featuring Tandem Ionization mass spectrometry

Marta Cialiè Rosso, Federico Stilo, Simone Squara, Erica Liberto, Stefania Mai, Chiara Mele, Paolo Marzullo, Gianluca Aimaretti, Stephen E. Reichenbach, Massimo Collino, Carlo Bicchi, Chiara Cordero

Step 1 - Untargeted Template Construction
Beginning with **48** chromatograms for parallel data streams (70, 12 eV) and for fused data (70+12 eV)

- Pre-processing: file import , rasterization, colorization
  baseline correction
  2D peaks detection and integration
- Comprehensive pair-wise peak matching
- Determination of *reliable* registration peaks (most relaxed constraint)
- Alignment of 2D chromatograms
- Generation of a composite chromatogram
- Definition of pattern of peak-regions for all detected 2D peaks
- Building of *feature* templates with *reliable* peaks and peak-regions

70 eV 12 eV fused data

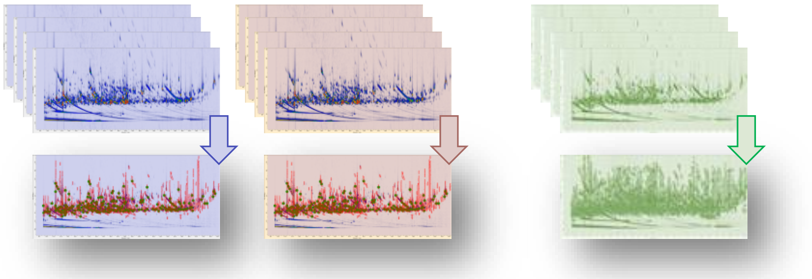

Step 2 - Cross-Sample Analysis
Feature templates built at Step 1 are matched on all sample images
- Template matching for *reliable* peaks and peak-regions
- Alignment of peak-regions relative to matched peaks
- Save processed chromatograms and export re-aligned metadata

Step 3 - Unsupervised and supervised chemometrics
Pattern recognition and variables selection
- Unsupervised PCA to examine natural conformation of groups
- Supervised exploration by PLS-DA and VIPs to define discriminant features between groups
- Cross-validation of data between detection channels
- Candidate peaks selection for targeting

Step 4 - Targeting of candidate markers and informative compounds
- Linear Retention Index (I^T) calibration
- Compound identification (NIST Algorithm)

Fig. S1 Detailed work flow applied for chromatographic fingerprinting of raw signals and Steps

A

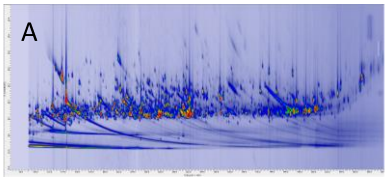

<u>70 eV data stream</u>
<u>Cumulative image - n° 2D peaks 1954</u>
Feature template
355 reliable peaks - green circles
454 peak-regions - red graphics

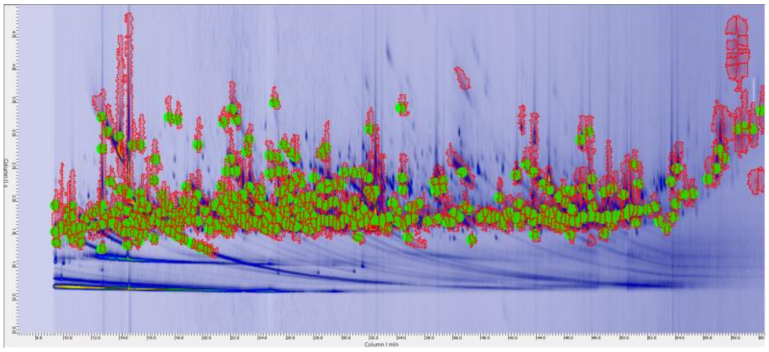

B

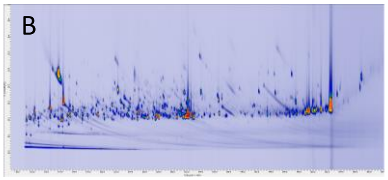

<u>12 eV data stream</u>
<u>Cumulative image - n° 2D peaks 1729</u>
Feature template
249 reliable peaks - green circles
521 peak-regions - red graphics

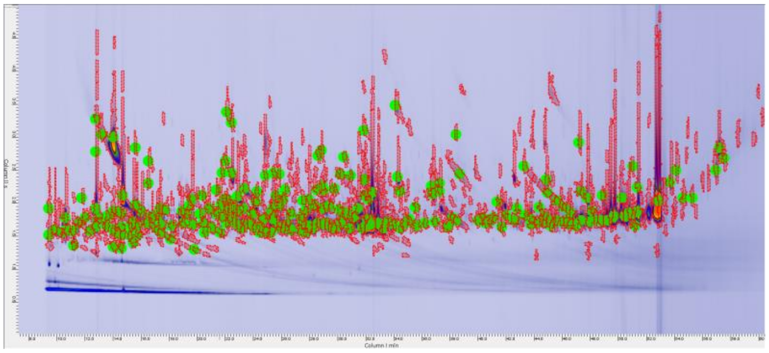

C

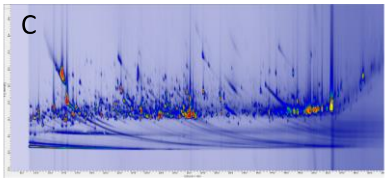

<u>fused data</u>
<u>Cumulative image - n° 2D peaks 2667</u>
Feature template
552 reliable peaks - green circles
1205 peak-regions - red graphics

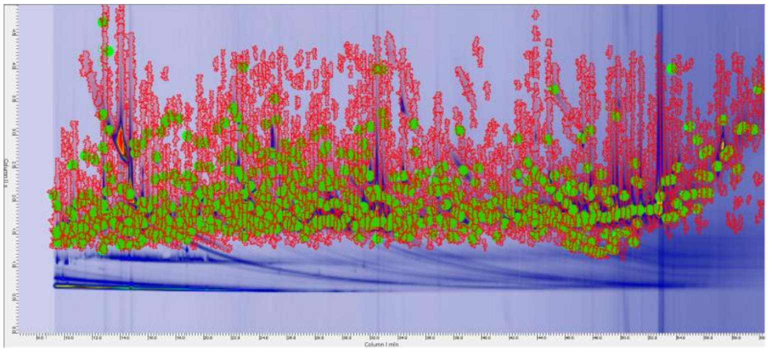

Fig. S2 Contour plots corresponding to cumulative images created by UT fingerprinting workflow (Figure 1) as they result by computing all available samples acquired at 70 eV ionization channel (SF1A), 12 eV (SF1B) and after fusion of the tandem data streams (SF2C). Green circles indicate reliable 2D peaks while red graphics indicate untargeted peak-regions. Details on feature template objects are also reported

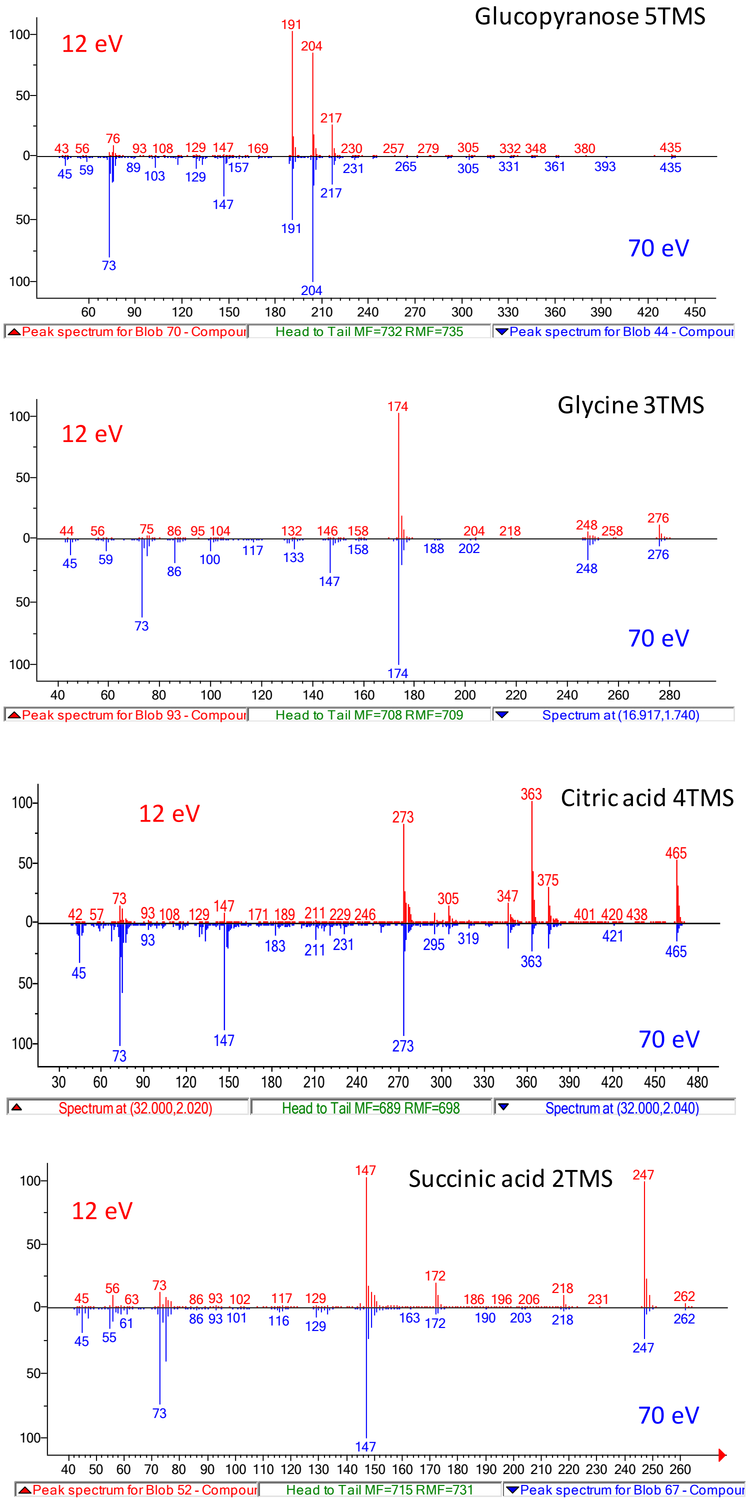

Fig. S3 Head-to-tail spectra obtained at 12 eV (red) and 70 eV (blue) for a selection of saliva metabolites

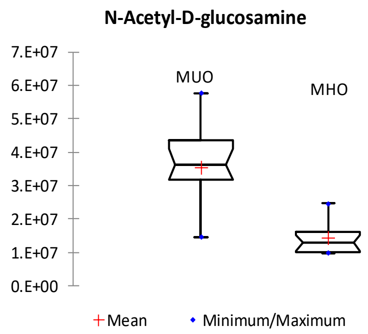

Statistic	MUO	MHO
No. of observations	20	12
Minimum	1.45E+07	9.85E+06
Maximum	5.76E+07	2.47E+07
1st Quartile	3.16E+07	9.93E+06
Median	3.63E+07	1.28E+07
3rd Quartile	4.35E+07	1.61E+07
Mean	3.54E+07	1.43E+07
Variance (n-1)	1.23E+14	2.33E+13
Standard deviation (n-1)	1.11E+07	4.82E+06

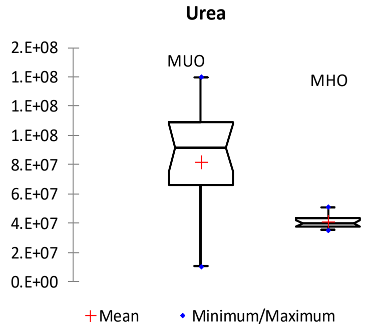

Statistic	MUO	MHO
No. of observations	20	12
Minimum	1.04E+07	3.51E+07
Maximum	1.40E+08	5.09E+07
1st Quartile	6.58E+07	3.76E+07
Median	9.18E+07	3.99E+07
3rd Quartile	1.09E+08	4.34E+07
Mean	8.16E+07	4.09E+07
Variance (n-1)	1.82E+15	2.27E+13
Standard deviation (n-1)	4.27E+07	4.76E+06

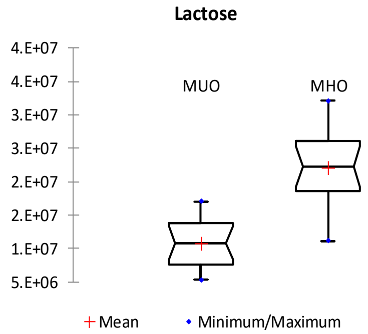

Statistic	MUO	MHO
No. of observations	20	12
Minimum	5.30E+06	1.11E+07
Maximum	1.71E+07	3.21E+07
1st Quartile	7.56E+06	1.86E+07
Median	1.08E+07	2.23E+07
3rd Quartile	1.38E+07	2.61E+07
Mean	1.07E+07	2.20E+07
Variance (n-1)	1.22E+13	3.43E+13
Standard deviation (n-1)	3.50E+06	5.85E+06

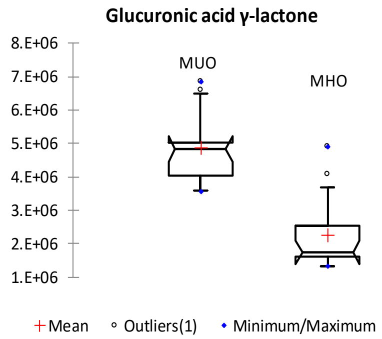

Statistic	MUO	MHO
No. of observations	20	12
Minimum	3.58E+06	1.33E+06
Maximum	6.87E+06	4.92E+06
1st Quartile	4.03E+06	1.60E+06
Median	4.83E+06	1.72E+06
3rd Quartile	5.03E+06	2.54E+06
Mean	4.88E+06	2.25E+06
Variance (n-1)	1.08E+12	1.16E+12
Standard deviation (n-1)	1.04E+06	1.08E+06

Fig. S4 Univariate statistics based on normalized TIC responses (over IS) for discriminant analytes

Table S1 Validation data

Feature #	Count	$^{1}t_R$			$^{2}t_R$			Count	QCs Corrected Percent Response		
		Mean	Stdev	%RSD	Mean	Stdev	%RSD		Mean	Stdev	%RSD
**(1)**	48	47.18	0.03	0.07	2.89	0.05	1.81	10	0.24	0.02	8.50
**(2)**	48	36.17	0.00	0.00	1.67	0.04	2.11	10	0.22	0.02	10.30
**(3)**	48	47.83	0.03	0.07	1.23	0.02	1.43	10	0.01	0.00	12.71
**(4)**	48	43.25	0.02	0.04	2.53	0.04	1.63	10	0.10	0.01	8.36
**(5)**	48	55.22	0.07	0.13	2.06	0.04	2.13	10	0.06	0.01	17.82
**(7)**	48	11.80	0.05	0.43	2.05	0.03	1.70	10	0.02	0.00	6.87
**(8)**	48	22.08	0.03	0.13	3.34	0.04	1.27	10	0.14	0.02	16.43
**(9)**	48	34.42	0.00	0.00	1.60	0.04	2.24	10	0.06	0.01	13.46
**(10)**	48	29.90	0.05	0.15	2.23	0.04	1.87	10	0.03	0.00	7.61
**(11)**	48	28.04	0.11	0.40	2.93	0.04	1.21	10	0.01	0.00	8.55
**(12)**	48	53.67	0.00	0.00	2.28	0.04	1.69	10	0.24	0.04	17.56
**(13)**	48	50.99	0.03	0.06	2.53	0.04	1.57	10	0.05	0.00	4.93
**(14)**	48	45.27	0.04	0.08	1.83	0.04	2.03	10	0.04	0.00	10.94
**(15)**	48	46.57	0.14	0.31	1.84	0.03	1.48	10	0.25	0.04	14.46
**(16)**	48	35.09	0.02	0.05	1.84	0.03	1.82	10	0.02	0.00	7.22
**(17)**	48	48.75	0.02	0.04	1.71	0.04	2.28	10	0.24	0.03	11.35
**(18)**	48	47.45	0.10	0.20	1.25	0.01	0.98	10	0.01	0.00	13.00
**(19)**	48	53.99	0.03	0.05	1.93	0.04	1.84	10	0.03	0.00	5.40
**(20)**	48	12.78	0.05	0.38	3.25	0.06	1.71	10	0.10	0.02	15.74
**(21)**	48	12.77	0.05	0.38	2.75	0.04	1.33	10	0.06	0.00	5.33
**(22)**	48	17.17	0.02	0.11	1.79	0.03	1.87	10	0.10	0.01	10.27
**(23)**	48	17.08	0.03	0.20	1.59	0.04	2.41	10	0.10	0.01	8.50
**(24)**	48	9.42	0.04	0.46	1.90	0.03	1.54	10	0.13	0.02	18.43
**(25)**	48	19.41	0.04	0.20	1.95	0.03	1.69	10	0.13	0.02	15.50
**(26)**	48	14.03	0.05	0.35	1.86	0.04	1.94	10	0.08	0.01	7.82
**(27)**	48	30.48	0.04	0.12	2.65	0.04	1.42	10	0.06	0.01	21.00
**(28)**	48	24.50	0.02	0.08	1.87	0.03	1.83	10	0.05	0.01	12.96
**(29)**	48	43.36	0.12	0.27	1.80	0.04	2.17	10	0.05	0.01	12.15
**(30)**	48	26.26	0.02	0.08	2.19	0.03	1.59	10	0.06	0.01	11.97
**(31)**	48	52.59	0.10	0.18	1.83	0.04	2.26	10	0.72	0.36	49.99
**(32)**	48	31.91	0.03	0.08	2.10	0.03	1.51	10	0.04	0.00	12.03
**(33)**	48	21.34	0.02	0.09	2.16	0.04	1.70	10	0.03	0.00	5.20
**(34)**	48	17.09	0.02	0.11	2.06	0.03	1.67	10	0.03	0.00	4.59
**(35)**	48	27.38	0.12	0.43	1.56	0.04	2.49	10	0.07	0.01	12.35
**(36)**	48	22.01	0.02	0.09	2.42	0.04	1.71	10	0.03	0.00	14.10
**(37)**	48	46.43	0.03	0.06	1.46	0.04	2.48	10	0.02	0.00	9.30
**(38)**	48	48.90	0.03	0.06	1.48	0.03	2.33	10	0.03	0.00	16.24
**(39)**	48	44.48	0.04	0.10	2.23	0.03	1.43	10	0.06	0.01	21.93
**(40)**	48	13.09	0.04	0.29	2.11	0.03	1.62	10	0.01	0.00	5.85
**(41)**	48	25.41	0.03	0.10	1.75	0.04	2.16	10	1.64	0.16	9.50
**(42)**	48	12.77	0.05	0.37	1.89	0.04	2.17	10	1.93	0.13	6.50
**(43)**	48	14.67	0.04	0.24	2.12	0.04	1.93	10	6.28	0.88	13.95
**(44)**	48	15.43	0.04	0.27	1.70	0.03	1.99	10	0.77	0.17	21.52

**(45)**	48	50.75	0.00	0.00	1.83	0.04	1.93	10	1.31	0.07	5.31
**(46)**	48	27.94	0.04	0.13	1.63	0.03	1.98	10	1.21	0.11	9.22
**(47)**	48	33.22	0.06	0.17	1.58	0.04	2.69	10	0.81	0.38	46.86
**(48)**	48	38.00	0.02	0.05	1.43	0.03	2.29	10	0.02	0.00	14.35
**(49)**	48	14.59	0.02	0.14	1.30	0.03	2.50	10	0.07	0.01	16.51
**(51)**	48	39.33	0.02	0.05	1.38	0.03	2.43	10	0.03	0.00	11.96
**(52)**	48	15.43	0.04	0.27	1.37	0.03	2.54	10	0.21	0.06	28.77
**(53)**	48	44.93	0.07	0.16	2.14	0.05	2.49	10	0.09	0.02	19.36
**(54)**	48	19.99	0.04	0.19	2.13	0.03	1.51	10	0.02	0.00	7.84
**(55)**	48	43.83	0.00	0.00	1.44	0.04	2.47	10	0.02	0.00	11.09
**(56)**	48	34.74	0.03	0.08	1.43	0.03	2.19	10	0.03	0.00	8.11
**(57)**	48	39.99	0.03	0.08	1.73	0.03	1.85	10	0.16	0.03	19.15
**(58)**	48	54.50	0.00	0.00	1.80	0.04	2.02	10	0.02	0.00	10.13
**(59)**	48	47.52	0.19	0.39	2.55	0.08	3.02	10	0.01	0.00	18.07
**(60)**	48	58.07	0.03	0.05	2.33	0.04	1.70	10	0.01	0.00	12.73
**(62)**	48	46.50	0.07	0.15	2.24	0.03	1.56	10	0.01	0.00	9.17
**(63)**	48	46.07	0.09	0.21	1.93	0.03	1.63	10	0.08	0.01	15.88
**(64)**	48	41.05	0.04	0.10	1.43	0.03	2.20	10	0.02	0.00	10.15
**(65)**	48	31.17	0.02	0.06	1.42	0.03	2.42	10	0.02	0.00	8.40
**(66)**	48	23.28	0.05	0.22	2.93	0.04	1.41	10	0.02	0.00	10.93
**(68)**	48	38.65	0.03	0.08	1.54	0.03	2.20	10	0.02	0.00	12.90
**(69)**	48	28.41	0.02	0.08	2.44	0.04	1.50	10	0.02	0.00	17.53
**(70)**	48	10.58	0.03	0.32	1.75	0.03	1.88	10	0.88	0.09	10.12
**(71)**	48	15.67	0.02	0.12	1.64	0.03	1.82	10	0.67	0.09	13.95
**(72)**	48	22.44	0.04	0.17	2.42	0.04	1.57	10	1.06	0.13	11.78
**(73)**	48	16.02	0.05	0.29	1.86	0.04	1.94	10	0.75	0.04	5.35
**(74)**	48	31.92	0.00	0.00	2.34	0.03	1.46	10	0.70	0.06	9.12
**(75)**	48	30.85	0.03	0.10	1.59	0.03	2.02	10	0.63	0.08	12.41
**(76)**	48	9.44	0.06	0.61	1.49	0.03	2.12	10	0.38	0.02	5.62
**(77)**	48	14.60	0.04	0.31	1.50	0.03	2.01	10	0.72	0.04	5.57
**(79)**	48	23.94	0.04	0.17	1.82	0.04	2.14	10	0.49	0.04	7.52
**(81)**	48	34.29	0.04	0.12	2.35	0.04	1.51	10	0.76	0.09	12.26
**(82)**	48	11.59	0.04	0.38	1.69	0.04	2.10	10	0.06	0.01	15.48
**(83)**	48	16.54	0.05	0.31	2.60	0.04	1.36	10	0.13	0.01	6.53
**(84)**	48	31.41	0.06	0.20	2.42	0.04	1.70	10	0.03	0.00	9.69
**(85)**	48	36.90	0.03	0.08	1.69	0.03	1.90	10	0.43	0.03	7.33
**(86)**	48	21.34	0.02	0.09	1.81	0.04	1.99	10	0.24	0.03	11.64
**(87)**	48	20.19	0.04	0.19	1.82	0.03	1.83	10	0.06	0.01	9.88
**(88)**	48	25.84	0.02	0.07	1.93	0.04	1.87	10	0.18	0.03	13.89
**(89)**	48	22.42	0.02	0.09	3.21	0.04	1.14	10	0.13	0.02	14.03
**(90)**	48	18.92	0.02	0.10	1.87	0.04	1.89	10	0.18	0.02	10.46
**(91)**	48	12.81	0.05	0.37	1.60	0.04	2.20	10	0.31	0.03	10.83
**(92)**	48	22.83	0.03	0.12	2.06	0.03	1.67	10	0.50	0.05	10.88
**(93)**	48	11.12	0.05	0.45	1.56	0.03	2.05	10	0.23	0.02	6.85
**(94)**	48	45.19	0.07	0.15	3.64	0.05	1.28	10	0.14	0.04	30.92
**(95)**	48	10.66	0.03	0.32	1.58	0.03	2.03	10	0.32	0.03	9.09
**(96)**	48	45.75	0.02	0.04	1.56	0.03	2.07	10	0.05	0.00	10.27

**(97)**	48	15.65	0.04	0.29	1.93	0.03	1.68	10	0.21	0.02	11.88
**(98)**	48	45.51	0.07	0.15	2.03	0.04	1.78	10	0.10	0.01	15.32
**(99)**	48	32.80	0.05	0.15	1.66	0.04	2.33	10	1.47	0.21	14.58
**(100)**	48	28.80	0.05	0.17	1.90	0.04	1.87	10	0.53	0.07	13.30
**(101)**	48	18.08	0.10	0.57	1.44	0.03	1.80	10	0.10	0.01	11.90
**(102)**	48	30.51	0.03	0.09	1.83	0.04	1.93	10	0.36	0.05	13.39
**(103)**	48	37.25	0.05	0.12	2.27	0.05	2.02	10	0.04	0.01	20.69
**(104)**	48	36.75	0.00	0.00	2.12	0.03	1.60	10	0.55	0.08	13.73
**(105)**	48	15.26	0.03	0.19	1.63	0.03	1.90	10	0.15	0.02	10.97
**(106)**	48	3.65	0.03	0.90	1.29	0.05	3.78	10	0.68	0.05	7.87
**(107)**	48	20.82	0.04	0.20	1.89	0.03	1.63	10	0.18	0.01	7.95
**(108)**	48	11.18	0.05	0.42	1.33	0.03	2.26	10	0.21	0.01	6.75
**(109)**	48	18.15	0.04	0.23	1.64	0.03	1.67	10	0.26	0.03	12.51
**(110)**	48	38.66	0.12	0.31	2.41	0.04	1.67	10	0.22	0.03	14.89
**(111)**	48	22.89	0.04	0.16	1.64	0.03	1.93	10	0.22	0.02	10.01
**(112)**	48	12.76	0.12	0.95	1.25	0.02	1.27	10	0.04	0.01	24.83
**(113)**	48	23.34	0.02	0.08	2.19	0.04	1.70	10	0.28	0.05	17.52
**(114)**	48	47.75	0.00	0.00	2.05	0.04	1.99	10	0.64	0.05	8.24
**(115)**	48	40.52	0.04	0.09	1.68	0.04	2.12	10	0.24	0.02	10.00
**(116)**	48	55.46	0.04	0.08	1.85	0.04	2.20	10	0.02	0.00	16.05
**(117)**	48	56.33	0.02	0.03	2.08	0.04	1.74	10	0.01	0.00	16.02
**(118)**	48	27.24	0.03	0.10	1.41	0.03	2.38	10	0.01	0.00	11.78
**(119)**	48	4.64	0.06	1.23	1.35	0.06	4.10	10	0.08	0.01	8.37
**(120)**	48	49.13	0.04	0.09	1.72	0.03	1.91	10	1.18	0.09	7.77
**(121)**	48	26.67	0.02	0.07	1.58	0.03	2.21	10	0.53	0.08	14.93
**(123)**	48	47.75	0.00	0.00	3.02	0.05	1.57	10	0.03	0.00	12.85
**(124)**	48	19.66	0.04	0.19	1.66	0.03	2.04	10	0.76	0.08	10.37
**(125)**	48	20.44	0.04	0.19	1.67	0.03	2.04	10	0.10	0.01	7.15
**(126)**	48	29.80	0.04	0.14	2.68	0.04	1.47	10	0.01	0.00	10.03
**(127)**	48	42.51	0.03	0.07	2.36	0.04	1.75	10	0.17	0.04	25.40
**(128)**	48	39.62	0.05	0.13	2.21	0.05	2.46	10	0.01	0.00	12.31
**(129)**	48	48.94	0.07	0.15	2.32	0.05	1.98	10	0.01	0.00	28.54
**(130)**	48	50.26	0.04	0.09	2.06	0.03	1.40	10	0.36	0.04	12.16
**(131)**	48	29.42	0.00	0.00	1.64	0.03	2.06	10	0.16	0.01	7.50
**(132)**	48	24.50	0.00	0.00	1.61	0.03	2.01	10	0.14	0.01	6.93
**(133)**	48	42.58	0.00	0.00	1.72	0.03	1.99	10	0.22	0.02	7.03
**(134)**	48	52.83	0.00	0.00	2.06	0.00	0.00	10	8.85	0.29	3.28
**(135)**	48	24.15	0.04	0.15	2.62	0.08	2.95	10	0.01	0.00	12.46
**(137)**	48	55.50	0.00	0.00	2.91	0.05	1.78	10	0.01	0.00	10.72
**(138)**	48	54.93	0.06	0.12	3.09	0.06	1.81	10	0.01	0.00	12.52
**(139)**	48	42.94	0.12	0.28	2.09	0.05	2.25	10	0.01	0.00	8.86
**(140)**	48	40.93	0.10	0.24	1.96	0.04	2.19	10	0.01	0.00	8.42
**(141)**	48	22.84	0.02	0.09	2.80	0.04	1.40	10	0.01	0.00	7.30
**(142)**	48	40.07	0.03	0.09	1.90	0.04	2.22	10	0.01	0.00	13.33
**(143)**	48	44.73	0.06	0.13	1.79	0.02	1.11	10	0.05	0.00	8.43
**(144)**	48	24.42	0.02	0.09	1.55	0.03	2.16	10	0.10	0.01	6.71
**(145)**	48	10.58	0.05	0.50	1.41	0.04	2.97	10	0.03	0.00	10.68

**(146)**	48	26.11	0.04	0.16	2.04	0.03	1.49	10	0.06	0.00	7.86
**(147)**	48	26.67	0.00	0.00	2.66	0.04	1.37	10	0.03	0.00	7.33
**(148)**	48	44.17	0.00	0.00	1.69	0.03	1.94	10	0.12	0.01	8.42
**(149)**	48	56.92	0.00	0.00	2.49	0.04	1.51	10	0.31	0.02	7.52
**(150)**	48	48.40	0.08	0.17	2.17	0.04	1.62	10	0.01	0.00	7.87
**(151)**	48	12.77	0.04	0.29	4.29	0.07	1.61	10	0.01	0.00	10.64
**(152)**	48	42.78	0.07	0.18	1.63	0.02	1.01	10	0.04	0.01	16.07
**(153)**	48	21.58	0.00	0.00	1.53	0.03	2.03	10	0.08	0.00	6.11
**(154)**	48	27.07	0.11	0.42	1.75	0.03	1.49	10	0.04	0.00	10.26
**(155)**	48	12.93	0.04	0.30	1.74	0.03	1.79	10	0.16	0.02	14.25
**(156)**	48	27.42	0.00	0.00	1.76	0.04	2.14	10	0.12	0.01	7.84
**(157)**	48	45.42	0.02	0.05	1.63	0.03	1.77	10	0.04	0.01	30.13
**(158)**	48	51.36	0.04	0.07	1.82	0.04	2.25	10	0.95	0.38	39.49
**(159)**	48	15.79	0.04	0.27	1.49	0.03	2.13	10	0.02	0.00	5.68
**(160)**	48	23.78	0.08	0.33	1.66	0.04	2.26	10	0.31	0.07	21.98
**(161)**	48	45.14	0.06	0.13	1.65	0.02	1.36	10	0.11	0.01	9.42
**(162)**	48	38.40	0.03	0.09	3.03	0.04	1.36	10	0.03	0.01	19.11
**(163)**	48	35.50	0.00	0.00	1.92	0.03	1.66	10	0.06	0.01	9.75
**(164)**	48	24.83	0.00	0.00	2.40	0.03	1.37	10	0.11	0.01	6.57
**(165)**	48	30.18	0.03	0.10	2.42	0.04	1.51	10	0.06	0.00	7.77
**(166)**	48	12.51	0.02	0.19	1.85	0.03	1.49	10	0.09	0.01	9.82
**(167)**	48	53.08	0.26	0.49	1.63	0.06	3.56	10	0.03	0.00	8.74
**(168)**	48	12.15	0.04	0.30	1.98	0.03	1.46	10	0.01	0.00	6.46
**(169)**	48	23.17	0.00	0.00	1.61	0.03	1.99	10	0.09	0.01	7.14
**(170)**	48	37.25	0.00	0.00	2.16	0.04	1.69	10	0.08	0.02	23.35
**(171)**	48	22.57	0.03	0.14	1.71	0.03	1.97	10	0.13	0.01	9.07
**(172)**	48	29.92	0.00	0.00	1.62	0.03	1.83	10	0.19	0.01	7.06
**(173)**	48	22.00	0.00	0.00	2.62	0.03	1.24	10	0.11	0.01	6.94
**(175)**	48	44.67	0.12	0.27	1.67	0.03	1.54	10	0.04	0.00	11.35
**(176)**	48	29.77	0.08	0.27	2.21	0.03	1.36	10	0.03	0.00	5.78
**(178)**	48	25.59	0.02	0.08	2.11	0.03	1.55	10	0.13	0.01	5.92
**(179)**	48	26.41	0.03	0.13	1.65	0.03	2.08	10	0.15	0.01	5.24
**(180)**	48	19.64	0.04	0.21	2.85	0.03	1.21	10	0.04	0.00	7.15
**(181)**	48	52.04	0.06	0.12	1.83	0.03	1.74	10	0.41	0.13	31.06
**(182)**	48	19.00	0.00	0.00	1.68	0.03	1.76	10	0.13	0.01	7.15
**(183)**	48	23.17	0.00	0.00	2.75	0.03	1.09	10	0.03	0.00	7.66
**(184)**	48	41.33	0.00	0.00	2.01	0.04	1.74	10	0.03	0.00	9.12
**(185)**	48	26.90	0.07	0.25	2.36	0.04	1.57	10	0.02	0.00	6.44
**(186)**	48	55.92	0.00	0.00	2.33	0.05	1.94	10	0.01	0.00	9.16
**(188)**	48	47.60	0.03	0.07	1.58	0.04	2.25	10	0.03	0.00	5.92
**(189)**	48	28.58	0.00	0.00	2.69	0.04	1.30	10	0.05	0.00	8.55
**(190)**	48	45.89	0.06	0.13	1.70	0.03	1.99	10	0.08	0.01	11.53
**(191)**	48	15.57	0.12	0.76	1.83	0.03	1.45	10	0.35	0.07	19.59
**(192)**	48	40.85	0.06	0.16	1.70	0.03	1.61	10	0.05	0.00	8.99
**(193)**	48	26.62	0.04	0.16	1.84	0.03	1.83	10	0.09	0.01	9.22
**(194)**	48	39.40	0.06	0.16	1.95	0.05	2.51	10	0.01	0.00	10.65
**(195)**	48	20.08	0.00	0.00	2.85	0.02	0.85	10	0.01	0.00	6.99

**(196)**	48	21.76	0.02	0.10	2.44	0.03	1.25	10	0.05	0.00	7.65
**(197)**	48	38.42	0.09	0.23	1.73	0.04	2.19	10	0.20	0.03	12.37
**(198)**	48	53.19	0.04	0.07	2.27	0.06	2.77	10	0.21	0.06	30.40
**(200)**	48	17.36	0.04	0.23	1.90	0.03	1.74	10	0.06	0.01	9.32
**(201)**	48	44.28	0.04	0.09	1.50	0.03	2.19	10	0.03	0.00	12.74
**(202)**	48	34.00	0.00	0.00	1.66	0.03	1.70	10	0.10	0.01	11.81
**(203)**	48	16.50	0.00	0.00	1.41	0.03	2.13	10	0.02	0.00	7.87
**(204)**	48	42.75	0.00	0.00	1.92	0.03	1.82	10	0.04	0.00	8.22
**(205)**	48	41.62	0.07	0.17	1.62	0.04	2.21	10	0.12	0.02	12.77
**(206)**	48	25.83	0.00	0.00	2.41	0.03	1.35	10	0.03	0.00	8.87
**(207)**	48	54.11	0.06	0.12	2.47	0.07	2.89	10	0.11	0.02	14.12
**(208)**	48	18.17	0.00	0.00	3.21	0.04	1.21	10	0.02	0.00	9.17
**(209)**	48	48.36	0.25	0.51	1.24	0.04	3.35	10	0.01	0.00	11.35
**(210)**	48	16.78	0.13	0.79	1.47	0.04	2.64	10	0.04	0.01	22.56
**(211)**	48	29.50	0.00	0.00	2.22	0.03	1.56	10	0.05	0.01	14.19
**(212)**	48	45.89	0.08	0.18	2.13	0.03	1.52	10	0.02	0.00	9.97
**(213)**	48	21.36	0.09	0.42	2.59	0.03	1.24	10	0.00	0.00	5.91
**(214)**	48	30.92	0.00	0.00	2.41	0.04	1.56	10	0.07	0.00	6.52
**(215)**	48	22.17	0.00	0.00	2.03	0.03	1.55	10	0.05	0.00	8.52
**(216)**	48	18.67	0.00	0.00	1.97	0.03	1.52	10	0.04	0.00	8.69
**(217)**	48	21.92	0.00	0.00	2.03	0.03	1.68	10	0.05	0.00	7.90
**(218)**	48	24.79	0.04	0.17	1.55	0.03	1.94	10	0.08	0.00	6.10
**(219)**	48	44.05	0.13	0.28	2.34	0.04	1.51	10	0.04	0.01	25.01
**(220)**	48	10.25	0.04	0.35	1.34	0.03	2.03	10	0.01	0.00	9.26
**(221)**	48	26.60	0.05	0.18	2.34	0.03	1.17	10	0.04	0.00	11.81
**(222)**	48	20.99	0.07	0.35	1.60	0.04	2.22	10	0.08	0.01	7.88
**(223)**	48	51.18	0.06	0.12	1.63	0.03	1.80	10	0.04	0.00	6.27
**(224)**	48	26.70	0.08	0.29	2.09	0.03	1.57	10	0.02	0.00	10.77
**(225)**	48	48.83	0.00	0.00	2.09	0.04	1.76	10	0.07	0.01	8.09
**(226)**	48	26.92	0.03	0.12	1.94	0.05	2.58	10	0.02	0.00	9.41
**(227)**	48	30.91	0.03	0.09	1.98	0.05	2.54	10	0.03	0.00	15.02
**(228)**	48	14.33	0.00	0.00	2.15	0.03	1.55	10	0.03	0.00	11.69
**(229)**	48	63.25	0.00	0.00	4.10	0.04	0.94	10	0.02	0.00	19.03
**(230)**	48	48.08	0.00	0.00	1.87	0.04	1.93	10	0.06	0.00	7.13
**(231)**	48	22.58	0.00	0.00	1.93	0.04	1.83	10	0.02	0.00	7.66
**(232)**	48	57.50	0.00	0.00	2.65	0.04	1.56	10	0.05	0.00	8.35
**(233)**	48	46.64	0.19	0.40	1.27	0.04	2.79	10	0.01	0.00	14.38
**(234)**	48	44.53	0.08	0.18	1.70	0.03	1.91	10	0.04	0.00	10.21
**(235)**	48	15.01	0.02	0.15	2.82	0.03	1.21	10	0.02	0.00	7.65
**(236)**	48	24.77	0.04	0.15	2.68	0.05	1.73	10	0.05	0.01	14.42
**(237)**	48	32.46	0.12	0.37	1.38	0.05	3.85	10	0.01	0.00	8.60
**(238)**	48	49.55	0.12	0.24	1.25	0.02	1.99	10	0.02	0.00	10.76
**(239)**	48	44.57	0.11	0.25	1.87	0.03	1.87	10	0.02	0.00	11.20
**(240)**	48	27.07	0.04	0.16	2.26	0.03	1.26	10	0.03	0.00	8.16
**(241)**	48	46.47	0.04	0.09	2.12	0.03	1.63	10	0.03	0.00	13.60
**(242)**	48	42.96	0.09	0.21	1.69	0.05	2.96	10	0.07	0.01	13.14
**(243)**	48	17.77	0.10	0.56	1.49	0.04	2.81	10	0.07	0.01	10.34

**(244)**	48	16.10	0.13	0.78	1.47	0.03	2.36	10	0.11	0.01	12.62
**(245)**	48	16.75	0.00	0.00	1.78	0.03	1.90	10	0.15	0.03	19.66
**(246)**	48	47.82	0.03	0.06	1.36	0.03	2.29	10	0.01	0.00	7.10
**(247)**	48	24.87	0.04	0.17	1.87	0.05	2.62	10	0.04	0.01	15.71
**(248)**	48	13.99	0.03	0.20	2.14	0.03	1.58	10	0.02	0.00	14.16
**(249)**	48	16.42	0.02	0.14	1.97	0.04	1.97	10	0.01	0.00	14.40
**(250)**	48	46.14	0.06	0.13	2.38	0.04	1.84	10	0.01	0.00	7.73
**(251)**	48	61.26	0.02	0.04	3.50	0.06	1.82	10	0.06	0.01	11.26
**(252)**	48	18.83	0.00	0.00	2.36	0.03	1.45	10	0.01	0.00	8.66
**(253)**	48	13.35	0.03	0.26	2.28	0.04	1.55	10	0.03	0.01	21.81
**(255)**	48	24.48	0.09	0.35	3.13	0.03	0.98	10	0.01	0.00	8.90
**(256)**	48	20.37	0.04	0.21	2.09	0.03	1.60	10	0.10	0.03	29.22
**(257)**	48	58.45	0.15	0.26	3.05	0.05	1.53	10	0.05	0.00	8.68
**(258)**	48	38.83	0.00	0.00	1.88	0.03	1.74	10	0.02	0.00	17.90
**(259)**	48	20.42	0.00	0.00	2.46	0.04	1.46	10	0.01	0.00	10.47
**(260)**	48	39.61	0.14	0.37	1.51	0.03	1.70	10	0.01	0.00	7.40
**(261)**	48	25.43	0.07	0.26	2.63	0.04	1.68	10	0.03	0.00	12.33
**(262)**	48	18.81	0.05	0.29	2.19	0.02	0.94	10	0.07	0.02	31.71
**(263)**	48	17.58	0.02	0.13	3.25	0.03	0.99	10	0.02	0.00	10.31
**(264)**	48	39.11	0.04	0.11	1.80	0.03	1.88	10	0.01	0.00	13.30
**(265)**	48	23.71	0.04	0.18	2.82	0.03	0.94	10	0.01	0.00	8.15
**(267)**	48	21.01	0.03	0.15	3.05	0.03	1.05	10	0.01	0.00	7.35
**(268)**	48	47.27	0.14	0.30	1.74	0.04	2.41	10	0.78	0.08	9.62
**(269)**	48	11.48	0.05	0.44	1.56	0.03	2.07	10	0.07	0.00	6.24
**(270)**	48	43.75	0.00	0.00	1.66	0.03	2.06	10	0.26	0.02	5.97
**(271)**	48	26.10	0.04	0.17	1.60	0.03	1.78	10	0.29	0.02	8.20
**(272)**	48	43.75	0.00	0.00	1.74	0.04	2.03	10	0.31	0.02	6.03
**(273)**	48	13.50	0.00	0.00	1.64	0.03	2.01	10	0.28	0.02	6.12
**(274)**	48	32.32	0.09	0.26	1.65	0.05	3.09	10	3.20	0.47	14.60
**(275)**	48	31.17	0.00	0.00	1.75	0.03	1.83	10	0.47	0.06	13.27
**(276)**	48	44.32	0.16	0.35	1.94	0.06	3.09	10	0.01	0.00	10.20
**(277)**	48	20.50	0.00	0.00	1.55	0.03	1.81	10	0.21	0.01	4.94
**(278)**	48	21.51	0.02	0.10	3.15	0.04	1.15	10	0.02	0.00	10.45
**(279)**	48	28.58	0.00	0.00	1.68	0.03	1.85	10	0.23	0.01	4.89
**(280)**	48	50.49	0.15	0.30	1.79	0.04	2.07	10	0.83	0.08	9.44
**(281)**	48	25.25	0.00	0.00	1.54	0.03	1.91	10	0.45	0.02	4.45
**(282)**	48	9.83	0.03	0.32	1.58	0.03	1.97	10	0.56	0.05	9.75
**(283)**	48	16.42	0.00	0.00	1.69	0.03	1.72	10	0.48	0.03	6.20
**(284)**	48	14.36	0.04	0.28	1.51	0.03	1.79	10	0.70	0.05	7.34
**(285)**	48	27.76	0.03	0.11	1.55	0.03	2.00	10	0.35	0.02	4.72
**(286)**	48	21.67	0.00	0.00	1.96	0.03	1.59	10	0.46	0.02	5.19
**(287)**	48	55.84	0.09	0.16	2.10	0.05	2.39	10	0.02	0.01	34.02
**(288)**	48	29.67	0.00	0.00	1.60	0.03	1.92	10	0.36	0.02	5.50
**(289)**	48	17.42	0.00	0.00	1.53	0.03	1.84	10	0.33	0.02	4.75
**(290)**	48	29.43	0.03	0.12	1.94	0.04	2.02	10	0.20	0.01	6.51
**(291)**	48	37.60	0.03	0.08	1.63	0.04	2.27	10	0.04	0.01	21.84
**(292)**	48	23.26	0.03	0.12	1.89	0.03	1.57	10	0.28	0.03	9.09

**(293)**	48	56.33	0.00	0.00	2.75	0.05	1.66	10	0.00	0.00	6.15
**(294)**	48	32.17	0.00	0.00	2.03	0.03	1.57	10	0.16	0.01	7.38
**(295)**	48	21.75	0.00	0.00	1.78	0.03	1.80	10	0.20	0.01	5.39
**(296)**	48	30.08	0.00	0.00	1.88	0.03	1.55	10	0.15	0.01	8.20
**(297)**	48	28.92	0.00	0.00	2.24	0.03	1.13	10	0.26	0.02	8.64
**(298)**	48	29.17	0.02	0.07	1.68	0.03	1.80	10	0.27	0.01	5.47
**(299)**	48	46.92	0.00	0.00	1.64	0.04	2.26	10	0.47	0.03	7.47
**(300)**	48	37.86	0.04	0.11	1.63	0.03	2.15	10	0.17	0.03	18.01
**(301)**	48	18.84	0.03	0.15	2.94	0.04	1.29	10	0.01	0.00	6.70
**(302)**	48	35.09	0.04	0.13	3.11	0.04	1.27	10	0.10	0.03	27.84
**(303)**	48	22.08	0.00	0.00	1.68	0.03	2.04	10	0.18	0.01	6.54
**(304)**	48	25.83	0.00	0.00	2.83	0.04	1.29	10	0.02	0.00	8.92
**(305)**	48	23.41	0.03	0.12	1.66	0.03	1.77	10	0.15	0.01	8.74
**(306)**	48	14.58	0.02	0.15	1.63	0.03	1.90	10	0.32	0.02	7.79
**(307)**	48	37.31	0.04	0.10	1.62	0.04	2.67	10	0.02	0.00	14.94
**(310)**	48	54.59	0.02	0.04	2.05	0.05	2.24	10	0.06	0.01	9.60
**(311)**	48	34.02	0.05	0.16	1.81	0.03	1.80	10	0.03	0.00	10.49
**(313)**	48	13.16	0.03	0.22	3.04	0.03	0.90	10	0.34	0.03	7.86
**(314)**	48	34.79	0.04	0.12	1.67	0.03	1.95	10	0.19	0.01	7.10
**(315)**	48	21.83	0.00	0.00	1.52	0.03	1.92	10	0.08	0.00	5.06
**(317)**	48	56.33	0.00	0.00	2.31	0.04	1.93	10	0.02	0.00	10.05
**(319)**	48	43.32	0.06	0.15	1.99	0.05	2.26	10	0.01	0.00	7.19
**(320)**	48	59.57	0.04	0.06	3.01	0.05	1.80	10	0.03	0.00	14.49
**(321)**	48	17.91	0.02	0.12	2.37	0.03	1.34	10	0.01	0.00	7.83
**(323)**	48	61.67	0.00	0.00	4.01	0.06	1.60	10	0.05	0.00	10.17
**(326)**	48	41.07	0.06	0.16	1.74	0.04	2.51	10	0.03	0.00	12.49
**(327)**	48	30.27	0.05	0.16	1.69	0.04	2.56	10	0.14	0.01	10.47
**(328)**	48	46.02	0.07	0.15	2.41	0.04	1.48	10	0.01	0.00	9.53
**(329)**	48	22.92	0.00	0.00	1.86	0.03	1.67	10	0.07	0.00	6.04
**(330)**	48	13.75	0.00	0.00	1.60	0.03	1.68	10	0.05	0.00	4.80
**(331)**	48	16.51	0.02	0.13	2.29	0.03	1.39	10	0.06	0.01	9.10
**(332)**	48	15.16	0.02	0.15	1.49	0.03	2.19	10	0.08	0.00	5.92
**(333)**	48	24.17	0.00	0.00	2.01	0.03	1.50	10	0.08	0.01	6.34
**(334)**	48	25.42	0.00	0.00	2.43	0.03	1.37	10	0.08	0.01	6.94
**(336)**	48	19.67	0.00	0.00	2.49	0.03	1.16	10	0.01	0.00	5.59
**(337)**	48	19.58	0.04	0.18	2.05	0.04	1.76	10	0.23	0.04	15.35
**(338)**	48	24.48	0.09	0.36	2.70	0.04	1.43	10	0.01	0.00	7.51
**(339)**	48	46.29	0.09	0.19	1.69	0.05	3.09	10	0.11	0.01	13.75
**(340)**	48	22.00	0.00	0.00	1.89	0.03	1.63	10	0.06	0.00	7.13
**(341)**	48	28.83	0.00	0.00	2.78	0.04	1.37	10	0.09	0.01	7.94
**(342)**	48	19.25	0.00	0.00	1.82	0.03	1.52	10	0.07	0.01	7.30
**(343)**	48	13.87	0.04	0.31	1.79	0.03	1.93	10	0.09	0.01	6.06
**(344)**	48	11.18	0.03	0.31	1.45	0.03	2.06	10	0.06	0.00	5.92
**(345)**	48	45.02	0.06	0.12	1.69	0.05	2.68	10	0.11	0.01	10.20
**(346)**	48	45.51	0.03	0.07	1.73	0.03	1.96	10	0.13	0.01	6.76
**(347)**	48	28.50	0.00	0.00	2.18	0.03	1.50	10	0.09	0.01	6.99
**(348)**	48	50.14	0.14	0.28	1.78	0.05	2.67	10	0.69	0.16	23.74

**(349)**	48	58.86	0.10	0.16	3.08	0.03	0.96	10	0.04	0.01	20.05
**(350)**	48	30.91	0.07	0.22	2.62	0.06	2.41	10	0.01	0.00	11.93
**(351)**	48	51.86	0.17	0.34	2.06	0.04	1.99	10	0.02	0.00	14.04
**(352)**	48	43.34	0.02	0.05	2.25	0.05	2.33	10	0.01	0.00	6.37
**(353)**	48	39.43	0.03	0.08	1.58	0.03	1.96	10	0.01	0.00	8.52
**(354)**	48	17.25	0.00	0.00	3.15	0.03	0.87	10	0.01	0.00	6.93
**(355)**	48	15.56	0.04	0.26	2.82	0.04	1.39	10	0.08	0.01	6.61
**(356)**	48	35.99	0.03	0.09	2.39	0.04	1.70	10	0.01	0.00	7.49
**(357)**	48	33.68	0.08	0.24	1.94	0.03	1.78	10	0.01	0.00	14.56
**(358)**	48	53.83	0.13	0.24	1.73	0.04	2.40	10	0.02	0.00	9.09
**(359)**	48	25.11	0.05	0.20	3.49	0.03	0.97	10	0.02	0.00	12.15
**(361)**	48	12.18	0.04	0.29	2.59	0.03	1.15	10	0.01	0.00	8.43
**(362)**	48	11.53	0.04	0.36	2.65	0.03	1.22	10	0.01	0.00	7.72
**(363)**	48	33.28	0.05	0.15	1.99	0.05	2.46	10	0.03	0.00	8.53
**(364)**	48	13.98	0.04	0.28	1.57	0.04	2.50	10	0.17	0.01	4.04
**(366)**	48	49.15	0.04	0.07	3.37	0.07	2.14	10	0.01	0.00	9.65
**(367)**	48	11.17	0.05	0.41	2.05	0.04	1.81	10	0.01	0.00	7.18
**(369)**	48	36.07	0.18	0.49	1.93	0.04	1.92	10	0.01	0.00	9.32
**(370)**	48	33.77	0.04	0.12	1.60	0.02	1.02	10	0.15	0.03	18.33
**(371)**	48	14.38	0.06	0.44	1.84	0.03	1.65	10	0.13	0.02	14.05
**(372)**	48	46.39	0.07	0.14	1.66	0.03	1.52	10	0.14	0.02	10.62
**(373)**	48	44.75	0.00	0.00	2.88	0.04	1.49	10	0.10	0.02	20.03
**(374)**	48	37.73	0.03	0.09	1.44	0.04	2.48	10	0.01	0.00	16.14
**(375)**	48	25.17	0.00	0.00	1.68	0.03	1.78	10	0.12	0.01	6.59
**(376)**	48	35.16	0.03	0.09	1.70	0.04	2.09	10	0.05	0.00	9.15
**(378)**	48	35.35	0.19	0.53	2.23	0.03	1.56	10	0.01	0.00	20.47
**(379)**	48	51.36	0.09	0.17	2.22	0.03	1.43	10	0.07	0.01	8.19
**(380)**	48	28.33	0.00	0.00	1.94	0.03	1.60	10	0.09	0.01	7.06
**(381)**	48	48.72	0.15	0.30	1.24	0.04	3.03	10	0.01	0.00	6.56
**(382)**	48	59.89	0.08	0.13	3.74	0.06	1.51	10	0.01	0.00	17.83
**(384)**	48	3.80	0.05	1.39	1.80	0.10	5.60	10	0.16	0.02	12.62
**(385)**	48	41.99	0.08	0.20	1.79	0.04	2.18	10	0.03	0.00	9.93
**(387)**	48	46.01	0.18	0.39	1.31	0.03	2.44	10	0.01	0.00	13.85
**(388)**	48	34.59	0.09	0.25	1.82	0.06	3.22	10	0.01	0.00	13.46
**(389)**	48	21.33	0.00	0.00	1.98	0.03	1.74	10	0.03	0.00	9.38
**(390)**	48	51.08	0.02	0.05	2.27	0.05	2.15	10	0.02	0.00	9.48
**(391)**	48	34.32	0.08	0.25	1.41	0.06	4.32	10	0.01	0.00	13.83
**(393)**	48	34.02	0.12	0.36	1.46	0.04	2.55	10	0.01	0.00	11.39
**(394)**	48	29.01	0.05	0.18	2.36	0.05	2.31	10	0.03	0.01	20.64
**(395)**	48	31.73	0.04	0.12	2.65	0.04	1.63	10	0.05	0.01	19.89
**(396)**	48	34.87	0.14	0.41	2.10	0.04	1.70	10	0.03	0.01	26.78
**(397)**	48	50.44	0.04	0.07	1.48	0.03	2.21	10	0.01	0.00	13.63
**(399)**	48	30.64	0.22	0.70	1.49	0.04	2.92	10	0.01	0.00	12.62
**(400)**	48	22.91	0.04	0.16	1.41	0.03	2.42	10	0.04	0.02	40.71
**(401)**	48	13.53	0.04	0.29	1.79	0.03	1.41	10	0.07	0.00	6.94
**(402)**	48	42.40	0.05	0.11	1.94	0.04	1.82	10	0.02	0.00	12.58
**(403)**	48	47.33	0.00	0.00	2.02	0.04	1.82	10	0.02	0.00	9.55

**(404)**	48	27.51	0.02	0.08	1.89	0.04	1.98	10	0.07	0.01	13.89
**(405)**	48	10.32	0.03	0.29	1.81	0.03	1.54	10	0.05	0.00	5.85
**(406)**	48	27.50	0.00	0.00	2.02	0.03	1.46	10	0.08	0.01	8.05
**(407)**	48	21.91	0.04	0.19	1.69	0.04	2.12	10	0.10	0.01	10.76
**(408)**	48	29.00	0.00	0.00	1.65	0.04	2.55	10	0.29	0.03	8.74
**(409)**	48	58.38	0.12	0.20	2.42	0.04	1.71	10	0.01	0.00	17.71
**(410)**	48	27.67	0.00	0.00	1.85	0.04	2.08	10	0.18	0.03	17.55
**(411)**	48	32.40	0.03	0.10	2.13	0.04	2.04	10	0.94	0.09	9.45
**(412)**	48	47.91	0.02	0.04	1.69	0.05	2.83	10	0.47	0.08	16.47
**(414)**	48	31.92	0.06	0.19	3.08	0.04	1.20	10	0.12	0.02	18.15
**(415)**	48	48.95	0.24	0.50	1.96	0.07	3.50	10	0.18	0.04	21.48
**(416)**	48	31.16	0.12	0.37	3.01	0.06	2.14	10	0.01	0.00	6.90
**(417)**	48	10.58	0.00	0.00	2.50	0.04	1.50	10	0.01	0.00	8.02
**(420)**	48	30.86	0.04	0.13	1.79	0.05	2.72	10	0.19	0.03	15.78
**(421)**	48	49.42	0.00	0.00	2.14	0.04	1.65	10	0.02	0.00	6.79
**(423)**	48	35.45	0.16	0.44	2.35	0.03	1.37	10	0.02	0.00	22.16
**(424)**	48	27.08	0.00	0.00	2.02	0.04	1.95	10	0.02	0.00	9.12
**(426)**	48	22.33	0.00	0.00	1.58	0.03	1.96	10	0.01	0.00	11.44
**(427)**	48	48.35	0.09	0.18	1.73	0.05	2.73	10	0.13	0.01	10.51
**(428)**	48	27.09	0.07	0.25	2.51	0.05	1.93	10	0.01	0.00	18.45
**(429)**	48	47.46	0.19	0.41	1.39	0.06	4.29	10	0.01	0.00	9.97
**(430)**	48	18.08	0.00	0.00	2.25	0.03	1.39	10	0.01	0.00	6.70
**(431)**	48	38.40	0.03	0.09	2.05	0.04	1.76	10	0.01	0.00	11.39
**(434)**	48	38.10	0.25	0.66	2.00	0.04	2.02	10	0.03	0.01	22.92
**(435)**	48	13.42	0.02	0.17	1.40	0.03	2.25	10	0.01	0.00	22.46
**(436)**	48	15.25	0.00	0.00	1.31	0.04	2.90	10	0.02	0.00	18.29
**(437)**	48	12.77	0.05	0.37	4.07	0.06	1.44	10	0.01	0.00	6.15
**(438)**	48	43.74	0.08	0.19	2.06	0.06	2.68	10	0.03	0.00	12.70
**(440)**	48	17.84	0.03	0.17	1.62	0.03	2.13	10	0.06	0.01	13.23
**(441)**	48	30.36	0.06	0.21	2.09	0.05	2.32	10	0.02	0.00	10.03
**(442)**	48	27.67	0.00	0.00	2.24	0.03	1.39	10	0.01	0.00	10.83
**(443)**	48	36.65	0.03	0.09	2.24	0.04	1.63	10	0.24	0.03	13.42
**(444)**	48	18.77	0.13	0.71	1.37	0.03	2.35	10	0.06	0.01	19.97
**(445)**	48	39.47	0.04	0.11	1.72	0.05	3.15	10	0.02	0.00	17.14
**(446)**	48	34.96	0.11	0.31	1.87	0.05	2.68	10	0.02	0.00	7.24
**(448)**	48	59.01	0.12	0.21	3.00	0.05	1.62	10	0.01	0.00	12.44
**(449)**	48	34.99	0.02	0.06	1.53	0.03	2.22	10	0.01	0.00	7.25
**(450)**	48	28.61	0.08	0.26	3.03	0.04	1.46	10	0.00	0.00	7.32
**(452)**	48	38.10	0.06	0.16	2.28	0.03	1.25	10	0.02	0.00	26.05
**(453)**	48	17.68	0.03	0.17	1.87	0.03	1.55	10	0.12	0.01	12.51
**(454)**	48	22.84	0.06	0.27	3.05	0.05	1.61	10	0.01	0.00	16.97
**(455)**	48	41.67	0.00	0.00	2.10	0.04	1.77	10	0.01	0.00	15.50
**(457)**	48	62.09	0.22	0.36	3.75	0.06	1.52	10	0.01	0.00	26.10
**(458)**	48	12.00	0.04	0.30	1.49	0.02	1.66	10	0.02	0.00	8.84
**(459)**	48	4.58	0.07	1.48	3.71	0.05	1.45	10	0.01	0.00	13.51
**(460)**	48	16.92	0.00	0.00	3.00	0.04	1.28	10	0.00	0.00	7.42
**(461)**	48	14.55	0.08	0.53	1.82	0.03	1.83	10	0.10	0.01	10.99

**(462)**	48	32.40	0.04	0.12	4.12	0.08	1.91	10	0.02	0.00	20.41
**(463)**	48	15.08	0.00	0.00	2.69	0.02	0.73	10	0.00	0.00	10.07
**(464)**	48	18.67	0.02	0.13	1.78	0.04	2.43	10	0.28	0.06	21.18
**(466)**	48	39.13	0.09	0.22	1.82	0.04	2.15	10	0.02	0.00	9.07
**(467)**	48	43.35	0.12	0.28	1.71	0.04	2.54	10	0.04	0.01	27.98
**(468)**	48	50.91	0.20	0.40	1.35	0.04	2.77	10	0.01	0.00	31.43
**(469)**	48	26.95	0.11	0.41	2.84	0.04	1.40	10	0.00	0.00	8.20
**(470)**	48	48.05	0.11	0.23	1.23	0.04	3.36	10	0.01	0.00	11.82
**(471)**	48	10.01	0.09	0.92	1.55	0.02	1.36	10	0.95	0.19	19.84
**(472)**	48	32.83	0.00	0.00	2.02	0.04	2.08	10	0.06	0.01	9.49
**(473)**	48	37.38	0.04	0.11	1.90	0.04	2.02	10	1.42	0.27	19.25
**(474)**	48	57.13	0.27	0.47	3.20	0.07	2.20	10	0.01	0.01	43.33
**(475)**	48	49.16	0.12	0.25	1.25	0.03	2.01	10	0.02	0.00	16.45
**(478)**	48	42.23	0.03	0.08	1.59	0.03	2.19	10	0.01	0.00	9.33
**(480)**	48	32.39	0.04	0.12	3.94	0.07	1.71	10	0.01	0.00	16.41
**(481)**	48	39.77	0.04	0.10	2.45	0.04	1.56	10	0.03	0.01	28.19
**(482)**	48	37.88	0.19	0.50	2.16	0.04	2.00	10	0.01	0.00	12.40
**(483)**	48	24.39	0.19	0.76	2.44	0.05	1.93	10	0.01	0.00	11.97
**(484)**	48	16.71	0.15	0.89	1.65	0.04	2.35	10	0.15	0.03	19.02
**(485)**	48	33.73	0.13	0.38	2.30	0.05	2.06	10	0.01	0.00	23.05
**(486)**	48	49.69	0.11	0.22	1.77	0.06	3.47	10	1.91	0.38	19.61
**(487)**	48	43.75	0.09	0.20	1.88	0.05	2.63	10	0.07	0.01	12.00
**(488)**	48	53.71	0.13	0.25	3.97	0.04	1.09	10	0.00	0.00	6.75
**(489)**	48	62.60	0.03	0.05	5.09	0.03	0.49	10	0.01	0.00	10.41
**(490)**	48	57.93	0.10	0.18	2.71	0.07	2.56	10	0.01	0.00	12.03
**(491)**	48	25.85	0.12	0.45	1.69	0.06	3.48	10	0.18	0.02	12.72
**(493)**	48	31.39	0.04	0.13	1.64	0.04	2.47	10	0.42	0.02	5.22
**(495)**	48	56.00	0.00	0.00	2.72	0.02	0.72	10	0.00	0.00	13.81
**(496)**	48	40.79	0.14	0.34	2.20	0.05	2.08	10	0.00	0.00	8.57
**(497)**	48	33.03	0.11	0.33	3.12	0.05	1.67	10	0.00	0.00	6.11
**(498)**	48	41.75	0.00	0.00	1.53	0.03	1.76	10	0.01	0.00	12.69
**(499)**	48	3.62	0.04	1.21	1.45	0.02	1.69	10	0.33	0.05	14.78
**(500)**	48	24.23	0.03	0.14	2.14	0.04	1.64	10	0.20	0.04	20.23
**(501)**	48	48.32	0.10	0.20	1.99	0.04	2.25	10	0.02	0.00	18.06
**(507)**	48	60.65	0.03	0.06	3.65	0.06	1.73	10	0.04	0.01	20.48
**(511)**	48	41.68	0.05	0.11	1.93	0.03	1.40	10	0.09	0.01	15.98
**(512)**	48	33.94	0.13	0.39	2.18	0.05	2.40	10	0.01	0.00	18.91
**(514)**	48	20.77	0.04	0.17	2.17	0.04	1.90	10	0.02	0.00	17.51
**(515)**	48	36.53	0.23	0.63	1.84	0.06	3.32	10	0.01	0.00	10.37
**(516)**	48	36.32	0.04	0.10	2.11	0.04	1.89	10	0.02	0.00	22.23
**(517)**	48	41.92	0.10	0.25	1.94	0.03	1.37	10	0.18	0.04	20.23
**(518)**	48	14.88	0.10	0.69	1.75	0.03	1.56	10	0.05	0.01	14.94
**(519)**	48	24.58	0.00	0.00	2.02	0.03	1.63	10	0.03	0.00	13.12
**(520)**	48	23.83	0.12	0.50	1.49	0.04	2.85	10	0.02	0.00	19.82
**(521)**	48	13.17	0.04	0.27	2.46	0.04	1.63	10	0.01	0.00	9.59
**(522)**	48	36.08	0.47	1.31	1.44	0.17	11.79	10	0.01	0.00	3.77
**(524)**	48	32.75	0.30	0.92	3.31	0.08	2.57	10	0.01	0.00	18.08

**(525)**	48	23.17	0.00	0.00	1.73	0.03	1.86	10	0.06	0.00	7.38
**(527)**	48	31.49	0.04	0.13	2.81	0.05	1.62	10	0.03	0.01	21.52
**(528)**	48	22.67	0.25	1.12	4.00	0.06	1.60	10	0.01	0.00	11.85
**(529)**	48	12.77	0.05	0.42	4.70	0.04	0.95	10	0.00	0.00	6.37
**(530)**	48	27.96	0.19	0.66	2.36	0.03	1.45	10	0.01	0.00	11.19
**(532)**	48	47.30	0.07	0.14	2.17	0.03	1.38	10	0.06	0.01	10.70
**(533)**	48	23.92	0.02	0.09	2.05	0.04	1.74	10	0.05	0.01	12.15
**(534)**	48	9.52	0.03	0.37	1.34	0.03	2.51	10	0.05	0.01	10.39
**(535)**	48	12.93	0.04	0.34	1.37	0.03	2.07	10	0.01	0.00	7.83
**(536)**	48	17.34	0.02	0.12	1.41	0.03	2.07	10	0.06	0.01	18.53
**(537)**	48	25.30	0.08	0.31	3.19	0.04	1.40	10	0.01	0.00	7.93
**(538)**	48	21.71	0.07	0.33	2.69	0.04	1.56	10	0.01	0.00	13.84
**(539)**	48	12.04	0.09	0.76	1.76	0.05	3.08	10	0.09	0.01	9.37
**(540)**	48	26.08	0.00	0.00	2.25	0.03	1.24	10	0.02	0.00	6.14
**(541)**	48	26.28	0.25	0.96	2.62	0.03	1.29	10	0.02	0.00	19.35
**(542)**	48	48.49	0.15	0.30	2.38	0.04	1.57	10	0.01	0.00	6.62
**(543)**	48	12.25	0.00	0.00	1.69	0.01	0.86	10	0.01	0.00	5.72
**(545)**	48	34.42	0.02	0.07	2.16	0.03	1.55	10	0.06	0.00	7.14
**(546)**	48	49.42	0.14	0.29	1.36	0.04	2.69	10	0.01	0.00	14.31
**(547)**	48	27.93	0.03	0.10	2.04	0.04	1.77	10	0.08	0.01	10.71
**(548)**	48	24.67	0.00	0.00	2.24	0.03	1.26	10	0.02	0.00	11.61
**(549)**	48	38.08	0.00	0.00	1.79	0.03	1.69	10	0.09	0.01	13.63
**(550)**	48	14.05	0.10	0.72	2.32	0.03	1.36	10	0.02	0.00	7.73
**(551)**	48	43.77	0.06	0.15	2.41	0.05	2.10	10	0.06	0.01	14.34
**(553)**	48	18.97	0.07	0.39	1.50	0.04	2.39	10	0.20	0.03	14.04
**(554)**	48	40.33	0.00	0.00	1.77	0.03	1.55	10	0.04	0.00	11.01
**(555)**	48	37.11	0.32	0.87	2.50	0.07	2.84	10	0.03	0.01	19.30
**(556)**	48	36.89	0.05	0.14	2.43	0.05	1.86	10	0.03	0.01	17.78
**(558)**	48	45.24	0.03	0.07	2.34	0.03	1.36	10	0.03	0.00	15.78
**(559)**	48	26.23	0.06	0.23	1.59	0.03	1.61	10	0.32	0.02	6.48
**(560)**	48	28.26	0.02	0.07	1.60	0.03	1.74	10	1.34	0.15	10.87
**(561)**	48	16.00	0.00	0.00	2.98	0.03	0.94	10	0.01	0.00	5.70
**(562)**	48	24.01	0.02	0.09	1.63	0.03	1.65	10	0.15	0.01	7.50
**(563)**	48	47.17	0.11	0.23	1.26	0.01	1.12	10	0.01	0.00	10.17
**(564)**	48	9.25	0.00	0.00	2.06	0.03	1.43	10	0.01	0.00	9.88
**(566)**	48	22.06	0.10	0.48	1.48	0.04	2.92	10	0.16	0.02	14.20
**(568)**	48	30.50	0.00	0.00	2.43	0.04	1.82	10	0.01	0.00	15.35
**(569)**	48	29.05	0.18	0.62	2.19	0.02	1.11	10	0.12	0.01	8.21
**(570)**	48	14.07	0.12	0.87	1.30	0.03	2.22	10	0.19	0.04	20.90
**(571)**	48	56.85	0.19	0.34	3.04	0.06	1.93	10	0.01	0.00	9.07
**(572)**	48	11.32	0.04	0.31	1.94	0.02	1.29	10	0.01	0.00	8.44
**(573)**	48	47.09	0.14	0.29	1.44	0.03	2.23	10	0.01	0.00	9.18
**(574)**	48	10.45	0.04	0.40	1.48	0.03	2.04	10	0.08	0.01	12.61
**(577)**	48	25.75	0.12	0.46	2.58	0.05	1.87	10	0.01	0.00	13.65
**(578)**	48	18.24	0.03	0.14	1.54	0.03	1.98	10	0.04	0.00	9.31
**(579)**	48	18.61	0.10	0.54	1.61	0.03	1.60	10	0.02	0.00	6.93
**(580)**	48	55.22	0.15	0.27	2.36	0.05	2.17	10	0.03	0.01	23.91

**(582)**	48	19.97	0.04	0.21	1.54	0.03	2.20	10	0.02	0.00	8.63
**(583)**	48	9.81	0.07	0.75	1.39	0.03	2.31	10	0.01	0.00	7.30
**(584)**	48	34.38	0.17	0.50	1.93	0.04	2.00	10	0.01	0.00	15.94
**(586)**	48	32.03	0.04	0.13	1.76	0.03	1.90	10	0.25	0.04	14.38
**(588)**	48	16.58	0.00	0.00	1.70	0.01	0.87	10	0.22	0.02	8.77
**(589)**	48	31.47	0.04	0.13	1.86	0.04	1.90	10	0.83	0.12	14.01
**(590)**	48	35.58	0.00	0.00	1.64	0.03	1.66	10	0.20	0.02	12.20
**(591)**	48	18.42	0.00	0.00	1.81	0.03	1.77	10	0.02	0.00	5.50
**(592)**	48	37.86	0.09	0.25	1.82	0.02	0.86	10	0.13	0.05	38.94
**(593)**	48	10.94	0.04	0.36	1.91	0.04	1.89	10	0.01	0.00	7.44
**(594)**	48	62.51	0.03	0.05	3.71	0.03	0.92	10	0.07	0.01	12.95
**(595)**	48	20.33	0.00	0.00	1.94	0.05	2.63	10	0.02	0.00	11.66
**(596)**	48	26.33	0.00	0.00	2.68	0.06	2.40	10	0.02	0.00	11.69
**(597)**	48	24.65	0.03	0.14	1.73	0.04	2.14	10	0.12	0.01	12.11
**(598)**	48	25.95	0.08	0.30	1.77	0.04	2.17	10	0.06	0.01	10.44
**(599)**	48	19.32	0.03	0.14	1.69	0.02	1.32	10	0.07	0.01	7.34
**(600)**	48	35.19	0.04	0.10	2.71	0.05	1.69	10	0.01	0.00	15.52
**(602)**	48	31.92	0.00	0.00	2.80	0.03	1.00	10	0.01	0.00	9.62
**(603)**	48	23.58	0.06	0.24	2.40	0.03	1.07	10	0.01	0.00	6.83
**(604)**	48	11.61	0.05	0.45	1.42	0.02	1.65	10	0.01	0.00	8.07
**(605)**	48	12.31	0.06	0.46	1.57	0.03	1.70	10	0.07	0.01	11.52
**(606)**	48	17.34	0.02	0.12	1.68	0.03	1.84	10	0.32	0.04	12.04
**(608)**	48	42.59	0.03	0.07	2.89	0.04	1.55	10	0.03	0.00	15.31
**(609)**	48	25.41	0.04	0.16	1.92	0.03	1.80	10	0.49	0.07	14.92
**(610)**	48	49.42	0.00	0.00	1.60	0.04	2.24	10	0.04	0.00	4.95
**(611)**	48	33.49	0.13	0.40	1.72	0.04	2.21	10	0.11	0.02	19.55
**(612)**	48	48.16	0.03	0.05	2.15	0.03	1.46	10	0.01	0.00	6.66
**(613)**	48	25.23	0.04	0.15	1.83	0.04	2.05	10	0.57	0.07	11.65
**(615)**	48	17.17	0.00	0.00	2.32	0.03	1.32	10	0.01	0.00	9.33
**(616)**	48	3.60	0.06	1.60	4.18	0.06	1.34	10	0.06	0.01	9.33
**(617)**	48	22.32	0.05	0.21	1.83	0.03	1.58	10	0.19	0.02	11.95
**(618)**	48	19.18	0.03	0.18	2.30	0.04	1.58	10	0.02	0.00	17.44
**(619)**	48	24.08	0.00	0.00	1.94	0.02	0.84	10	0.09	0.00	4.60
**(620)**	48	31.75	0.00	0.00	1.59	0.03	1.73	10	0.66	0.11	16.49
**(621)**	48	22.85	0.03	0.14	2.32	0.03	1.45	10	0.03	0.01	15.62
**(622)**	48	40.50	0.05	0.13	1.88	0.04	1.93	10	0.02	0.00	14.50
**(623)**	48	46.60	0.13	0.28	1.75	0.05	2.64	10	0.15	0.03	18.69
**(624)**	48	16.42	0.06	0.39	1.83	0.04	2.15	10	0.03	0.00	8.23
**(625)**	48	23.33	0.00	0.00	2.37	0.05	1.98	10	0.03	0.01	22.08
**Average value**				**0.20**			**1.90**				**12.13**
**Min**				**0.00**			**0.00**				**3.28**
**Max**				**1.60**			**11.79**				**49.99**
